# A family of linear plasmid phages that detect a quorum-sensing autoinducer exists in multiple bacterial species

**DOI:** 10.1128/mbio.02320-25

**Published:** 2025-12-12

**Authors:** Francis J. Santoriello, Bonnie L. Bassler

**Affiliations:** 1 Department of Molecular Biology, Princeton University 6740, Princeton, New Jersey, USA; 2 Howard Hughes Medical Institute 2405, Chevy Chase, Maryland, USA; University of Pittsburgh, Pittsburgh, Pennsylvania, USA

**Keywords:** quorum sensing, vibrio, bacteriophage, linear plasmid phage, phage-bacterial communication

## Abstract

Temperate phages oscillate between lysogeny, a genomic maintenance state within a bacterial host, and lytic replication, in which the host is killed, and newly made phage particles are released. Successful transmission to new hosts requires that temperate phages appropriately time their transitions from lysogeny to lysis. It is well understood that temperate phages trigger lysis upon detection of host cell stress. Understanding of the breadth of cues that induce lysis expanded with the discovery of phages carrying quorum-sensing receptor genes that promote lytic induction exclusively at high host cell density. Bacteria engage in a cell-cell communication process called quorum sensing, which relies on the production, release, accumulation, and group-wide detection of extracellular signal molecules called autoinducers. Bacteria use quorum sensing to monitor changes in population density and synchronize collective behaviors. The temperate phage VP882 (φVP882) encodes VqmAφ—a homolog of its host’s quorum-sensing receptor/transcription factor VqmA. VqmAφ allows φVP882 to detect the accumulation of the host autoinducer called DPO. Presumably, launching the lytic induction program at high host cell density maximizes φVP882 transmission to new hosts. Here, by mining sequence databases for linear plasmid phages, we identify VP882-like phages in multiple DPO-producing bacterial species isolated at diverse times and geographic locations. We show that the VqmAφ homologs can indeed detect DPO and, in response, activate the lytic pathway. Our findings indicate that φVP882 is a member of a family of globally dispersed temperate phages that possess the potential to respond to host quorum sensing.

**IMPORTANCE:**

The discovery of quorum-sensing responsive linear plasmid phages has transformed understanding of phage-bacterial interactions by demonstrating inter-domain chemical communication. To date, however, examples of quorum-sensing responsive phages have been sparse. The founding example of such a phage, φVP882, detects a chemical communication signal molecule called DPO that is produced by diverse bacterial species. We investigated whether a family of VP882-like phages might exist that detect and respond to DPO. We find that indeed, VP882-like phages reside in DPO-producing bacterial species isolated at different times and geographic locations, suggesting their wide circulation in the environment. This discovery strengthens the evidence for the generality of phage-bacterial inter-domain chemical communication.

## INTRODUCTION

Bacteria monitor the cell density and species composition of their vicinal environments using a process called quorum sensing, which involves the production, release, accumulation, and detection of extracellular molecules called autoinducers. Relevant to the present work is one such quorum-sensing system, called VqmAR, that is conserved across vibrios (1, 2)—a genus of bacteria that includes quorum-sensing model organisms such as *Vibrio fischeri*, *Vibrio campbellii*, *Vibrio cholerae*, and *Vibrio parahaemolyticus*. In VqmAR-directed quorum sensing, at high cell density, VqmA, which is a cytoplasmic receptor/transcription factor, binds its cognate autoinducer 3,5-dimethyl-pyrazin-2-ol (DPO), and activates expression of the gene encoding a small RNA (sRNA) called VqmR. VqmR functions by a post-transcriptional mechanism to control the expression of genes underpinning group behaviors (1, 3, 4). The DPO autoinducer is made from threonine and alanine, and production requires the threonine dehydrogenase (Tdh) enzyme (3, 4). Tdh is highly conserved across bacterial species (NCBI HMM: TIGR00692.1). Thus, any bacterium encoding a functional Tdh has the potential to produce DPO, indicating the capacity of DPO to serve as a broadly used autoinducer. Despite the breadth of DPO production, the vibrio-specific quorum-sensing receptor VqmA is the only known DPO-binding receptor.

DPO is also involved in bacteria-virus chemical communication. The *V. parahaemolyticus* temperate, linear plasmid phage VP882 (φVP882) monitors host quorum-sensing-mediated communication using a phage-encoded homolog of VqmA, denoted VqmAφ (2). Analogous to VqmA, at high host cell density, the VqmAφ receptor binds host-produced DPO. DPO-bound VqmAφ triggers transcription of a small open reading frame (smORF) encoding a phage antirepressor, Qtip. Qtip sequesters and inactivates the phage cI lysis repressor to initiate lytic replication (2, 5). We hypothesize that this host-DNA-damage-independent pathway to lytic induction allows φVP882 to coordinate lysis with a high vicinal density of potential new host cells, thus maximizing transmission. Since the discovery of the VqmAφ-Qtip transcription factor-small open reading frame (TF-smORF) module, other linear plasmid phages with different TF-smORF modules have been identified (6).

Interactions between φVP882 and its *V. parahaemolyticus* host are complex, as other vibrio quorum-sensing systems, including the canonical LuxO-OpaR system, have been shown to affect φVP882 lysis-lysogeny transitions (7, 8). φVP882 also potentially uses VqmAφ to influence host gene regulation; host VqmA can only bind and activate the host *vqmR* promoter, but VqmAφ can bind and activate both the host *vqmR* promoter and the phage-borne *qtip* promoter (9). The regulatory relationships between φVP882 and *V. parahaemolyticus* are apparently only applicable to the vibrio genus due to restriction of the involved host quorum-sensing machinery to vibrios (2). By contrast, both DPO production and lysogeny by linear plasmid phages are widespread in the bacterial domain (6, 10). We hypothesize that any VP882-like linear plasmid phage harboring a VqmAφ-Qtip system could surveil and respond to the cell density of a DPO-producing host bacterial species. Despite this potential for broad pertinency, VP882-like phages initially appeared to be rare in sequence databases. φVP882 was first identified in *V. parahaemolyticus* strain 882 (11). Over a decade later, two VP882-like phages were identified, one in a different *V. parahaemolyticus* strain and one in a *Salmonella enterica* strain (5). Since that work, efforts to catalog bacteriophages in various environments have expanded sequence databases (12–16). Considering the breadth of DPO production among bacteria, we searched these enlarged sequence repositories for additional VP882-like phages carrying *vqmAφ-qtip*. Here, we show that VP882-like phages are harbored by diverse bacterial species that have been isolated from broadly varying environmental niches in widespread geographic locations over a span of 26 years. We demonstrate that the DPO-VqmAφ-Qtip circuits are intact and functional in VP882-like phages across a variety of hosts. These results suggest that VP882-like phages constitute a closely related family of phages circulating globally in diverse bacterial species.

## RESULTS

### Datamining for linear plasmid phages identifies 22 closely related VP882-like phages

**Fig 1:**
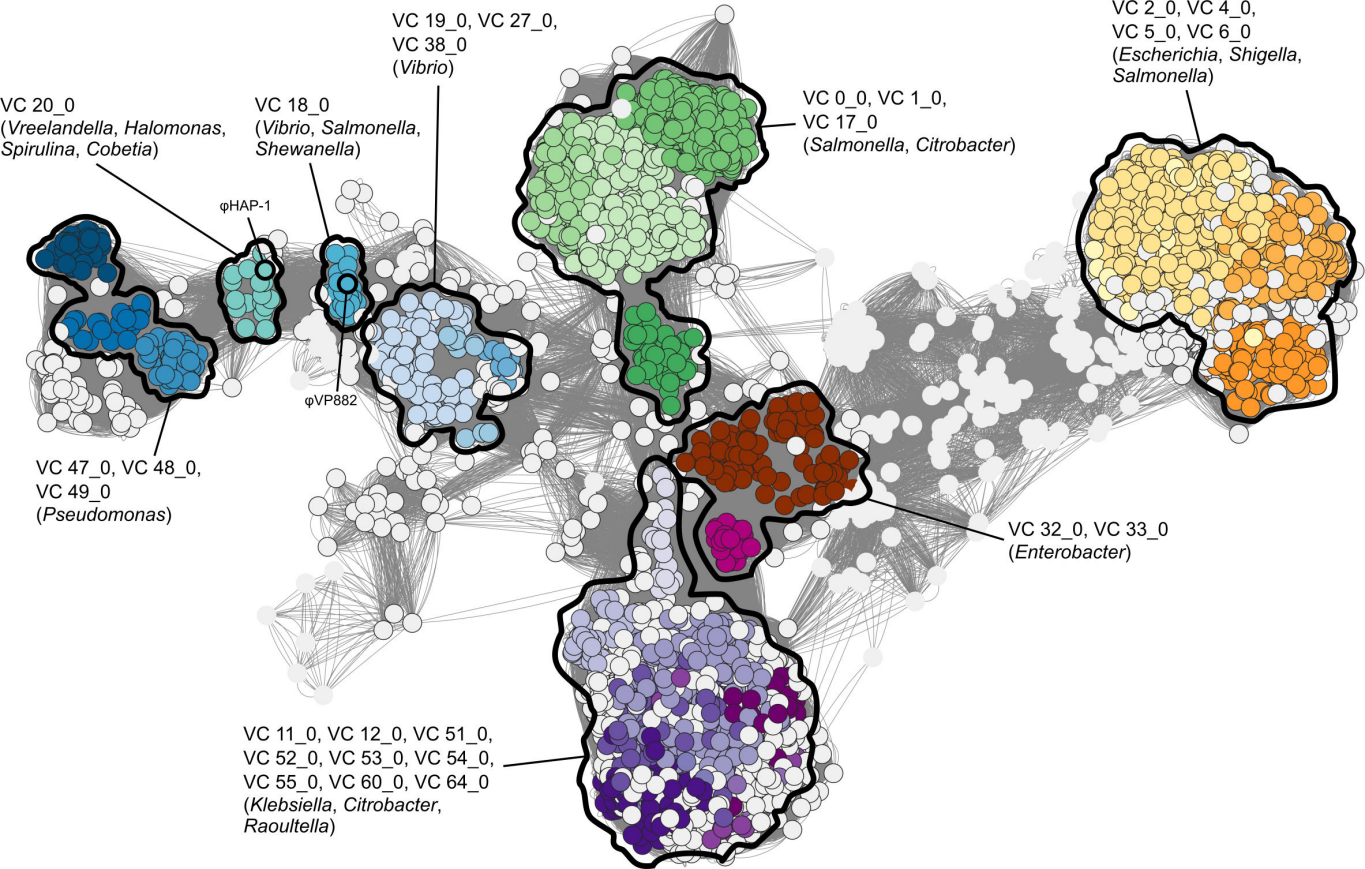
φVP882 clustered with VP882-like linear plasmid phages that lysogenize diverse bacterial species. Taxonomic network of collected linear phage genomes against RefSeq viral sequences. Nodes representing linear phages collected from the six queried databases are outlined in black. Nodes from the Prokaryotic Viral RefSeq database are not outlined. Edge weights were calculated by vConTACT2. Viral clusters with more than 20 nodes are colored. Individual clusters or groups of clusters are outlined and labeled with their respective VC numbers and the most abundant associated genera.

We used TelN, a conserved component of the linear plasmid phage replication machinery (17), to query NCBI and five phage-specific databases for linear plasmid phage sequences (12–16). We extracted 8,537 unique phage sequences (Data set S1). Most sequences (8,148) in this data set were from NCBI, and of these, the predominant host genera were *Klebsiella* (3,151), *Escherichia* (1,992), *Salmonella* (1,911), *Shigella* (419), *Pseudomonas* (213), *Enterobacter* (168), and *Vibrio* (124). The remaining 389 non-NCBI sequences were extracted from the IMG/VR, CHVD, GOV2, GPD, and MGV databases (12–16). We clustered the identified phages with the Prokaryotic Viral RefSeq database to identify their genus-level relationships with annotated RefSeq viral sequences. The analysis returned 838 viral clusters (Fig. 1; Data set S2), of which 79 were comprised of linear plasmid phages. Reflecting the host species distribution of the mined genomes, the majority of the 79 viral clusters consisted of large groups of closely related phages that infect a single host genus, typically *Klebsiella*, *Escherichia*, or *Salmonella* (Fig. 1). This trend was previously demonstrated for the *Klebsiella* NAR688-like linear plasmid phages (10). We focused on φVP882, which clustered into VC_18_0 with 19 other phage genomes from at least six host species: *V. parahaemolyticus*, *Vibrio vulnificus*, *V. cholerae*, *S. enterica*, *Shewanella algae*, and a *Rikenellaceae* family isolate (Fig. 1; Table S1; Data set S2). This cluster returned a viral Genus Confidence Score of 1.0, indicating that all phages in the cluster are likely of the genus *Hapunavirus*, which prior to our analysis, only included φVP882 and φHAP-1 of *Vreelandella aquamarina* (18). Our analysis shows that, in fact, φHAP-1 clusters into VC_20_0 along with 18 other phage genomes (Fig. 1; Data set S2), indicating that HAP-1-like hapunaviruses are distinct from VP882-like hapunaviruses (Fig. S1). Unlike φVP882, none of the HAP-1-like phages encode VqmAφ-Qtip or any other TF-smORF module (Fig. S1).

**Fig 2:**
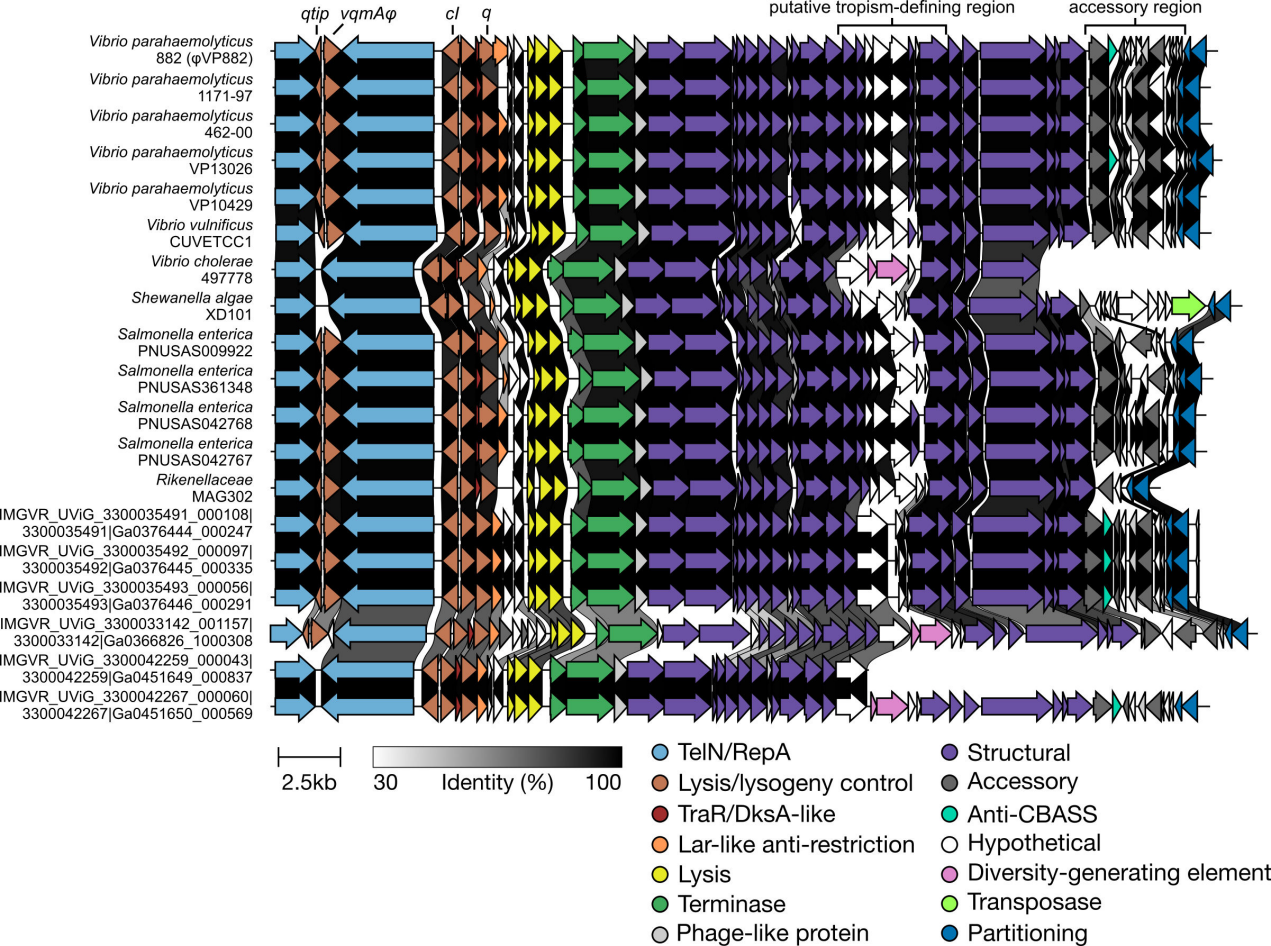
φVP882-like phages comprise a closely related family of putative quorum-sensing-responsive linear plasmid phages. Genome synteny of VP882-like linear plasmid phages. Host species and strain are provided on the left. Sequences designated IMG/VR were collected from metagenomic data and thus, do not have associated host strains. Arrows represent genes colored according to their annotated functions. Gene homologs in neighboring sequences are connected by shaded links. Shading represents the % identity between the amino acid sequences of the proteins encoded by the homologous genes. The absence of a link indicates less than 30% amino acid identity between proteins encoded by neighboring genes or the absence of a homolog in the neighbor.

**Fig 3:**
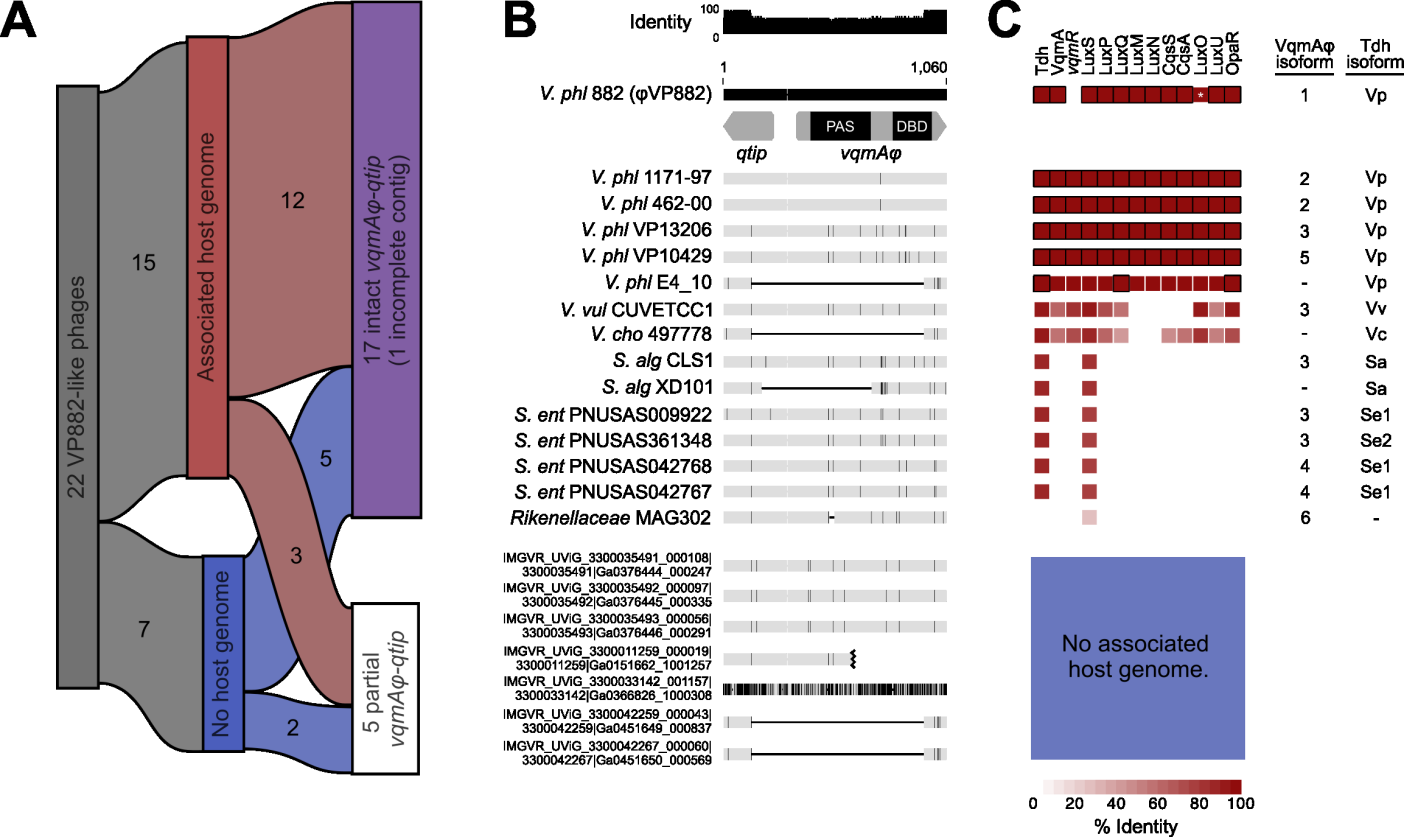
The *vqmAφ-qtip* module is highly conserved across VP882-like linear plasmid phages. (**A**) Chart showing subsets of VP882-like phages discussed in the text. (**B**) Nucleotide alignment of the genome region encoding the *qtip* and *vqmAφ* genes from 22 VP882-like phages. A schematic of *qtip* and *vqmAφ* is shown for the uppermost sequence, and the regions encoding the VqmAφ DNA-binding (DBD) and ligand binding (PAS) domains are indicated. The host species and strain are provided on the left (*V. phl* = *Vibrio parahaemolyticus*, *V. vul* = *Vibrio vulnificus*, *V. cho* = *Vibrio cholerae*, *S. alg* = *Shewanella algeae*, and *S. ent* = *Salmonella enterica*). Sequences designated IMG/VR were collected from metagenomic data and thus do not have associated host strains. Gray horizontal bars represent the homologous DNA sequences, and black vertical lines within the gray bars represent nucleotide differences relative to *qtip-vqmAφ* from the φVP882 reference sequence. Thin black horizontal lines denote deleted DNA sequences. The black jagged mark in the IMG/VR sequence ending with Ga0151662_1001257 denotes the terminus of the contig. (**C**) Heat map of quorum-sensing protein amino acid identity across host strains carrying VP882-like phages. Identity was calculated relative to quorum-sensing proteins from *V. parahaemolyticus* type strain RIMD 2210633. Black outlines denote 100% identity. Blank spaces indicate no detected homolog. The white asterisk in LuxO from *V. parahaemolyticus* 882 indicates the constitutive low-cell-density-locked status of the protein, despite high homology, due to a 12 amino acid deletion relative to the type strain. Pairs of VqmAφ and Tdh isoforms for each strain are listed to the right of the heatmap.

We aligned the genomes of 19 of the 20 VP882-like phages (one partial genome was excluded) and identified conservation over their entire genomes apart from two variable regions (Fig. 2): one in the structural gene region that encodes a predicted P1-like GpU (UniProt: Q71TD6 · U1_BPP1), likely involved in host tropism, and one covering the accessory region, a common site of genomic innovation (19). To the 20 clustered VP882-like phages (Fig. 1), we added two more putative VP882-like phage genomes, harbored by *V. parahaemolyticus* E4_10 and *S. algae* CLS1 (Table S1). These genomes were initially excluded from our search due to their fragmented natures; however, they carry either a complete *vqmAφ-qtip* module (*S. algae* CLS1) or fragments of *vqmAφ* and *qtip* (*V. parahaemolyticus* E4_10). The quorum-sensing-responsive *vqmAφ-qtip* module is conserved in 17 of the 22 VP882-like phages (Fig. 3A and B). We note that 1 of the 17 VP882-like phages harbors the *vqmAφ-qtip* module at the end of its contig, and thus it cannot be verified as intact (Fig. 3A and B). The five phages lacking the module possess fragments of both the *vqmAφ* and *qtip* genes, indicating elimination of a portion of the module, presumably via deletion (Fig. 3A and B). Among the 16 VP882-like phages with a complete *vqmAφ-qtip* module, polymorphisms have yielded nine VqmAφ isoforms that do not cluster by host species (denoted VqmAφ^1–9^ in Fig. S2A). The Qtip proteins are identical in 14 of the 16 genomes (Fig. S2B). The partner cI proteins to the 14 identical Qtip proteins differ from the φVP882 cI protein by 0–11 amino acid substitutions and insertions (Fig. S2C). This finding suggests that the 14 identical Qtip proteins can tolerate these variations. The two non-identical Qtip proteins co-occur with more distant cI proteins (68% and 44% amino acid identity to the φVP882 cI; Fig. S2B and C).

### Bacterial strains harboring VP882-like phages have the capacity to produce DPO

Of the 22 VP882-like phages, 15 had an associated host genome (Fig. 3A through C). The remaining seven were extracted from metagenomic databases and thus lack a corresponding host genome sequence (Table S1). For the remainder of this study, we focus on the 15 VP882-like phages that have associated host genomes. These VP882-like phages were identified in six *V*. *parahaemolyticus* strains, one *V. vulnificus* strain, one *V. cholerae* strain, four *S*. *enterica* strains, two *S*. *algae* strains, and one *Rikenellaceae* family strain (Fig. 2; Table S1). In *V. parahaemolyticus*, φVP882 lysis-lysogeny transitions are influenced by VqmAφ-Qtip, host DPO production, and the vibrio host LuxO-OpaR quorum-sensing system (7, 8). With the aim of assessing the capacity of each host bacterial species identified here to interact with the VP882-like phages it carries, we determined the presence or absence of vibrio quorum-sensing genes in the hosts (Fig. 3C; Fig. S3A through E). Nearly all vibrio quorum-sensing genes are restricted to vibrio hosts. The exceptions are *tdh* (present in 14/15 hosts) and *luxS* (present in 15/15 hosts) (Fig. 3C; Fig. S3A and D). The single host genome lacking a *tdh* gene, *Rikenellaceae* MAG302 (Fig. 3C), is a contig-level assembly, and so we cannot be certain that it indeed lacks *tdh*. Tdh and LuxS produce the universal autoinducers DPO and AI-2, respectively (3, 20). We presume that only DPO binds VqmAφ to trigger Qtip-driven lytic induction (2). Thus, here we focus on Tdh, which was identified as present in all but one VP882-like phage host strain (Fig. 3C).

**Fig 4:**
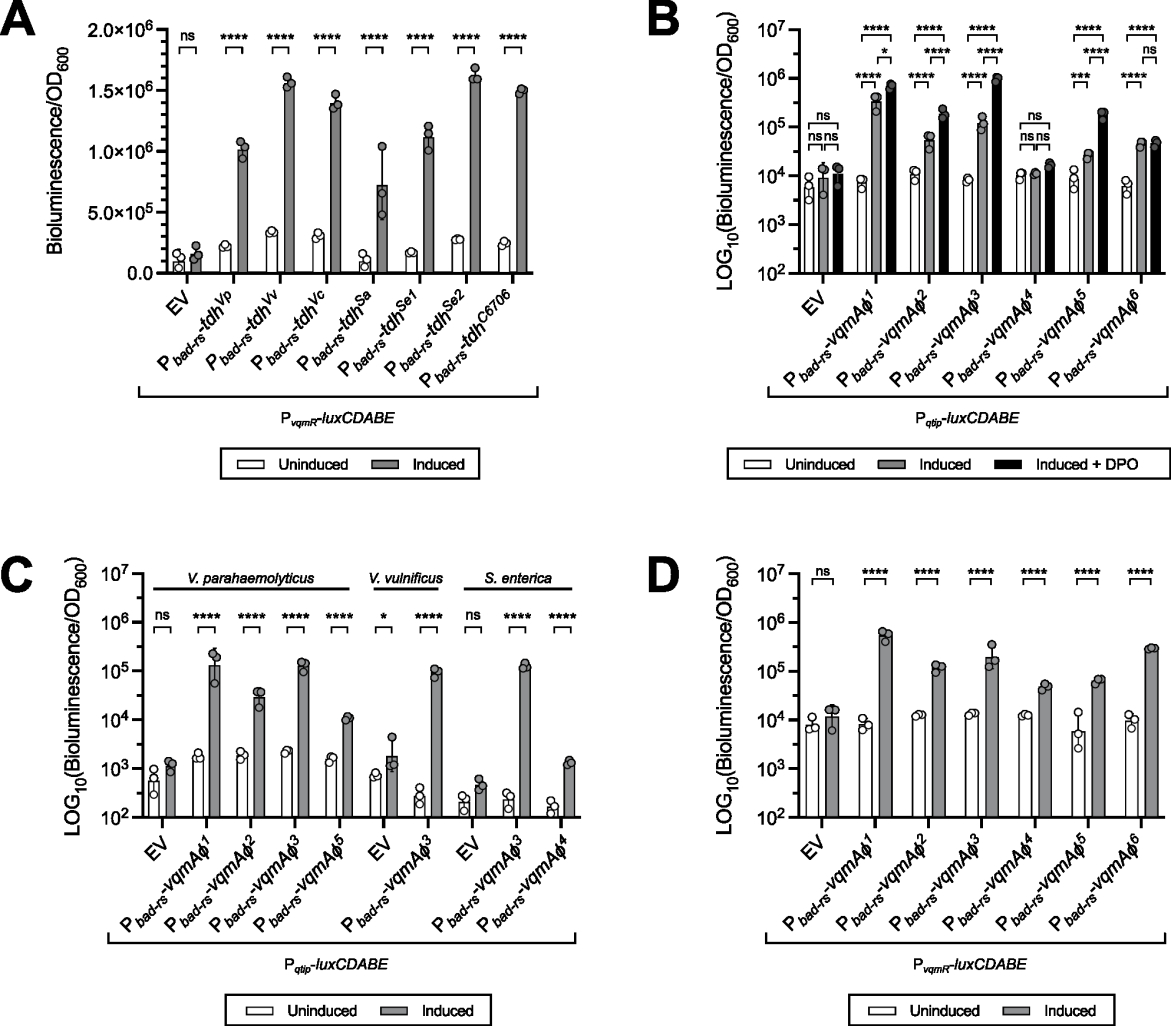
The DPO-VqmAφ-Qtip circuit is functional in most VP882-like phage lysogens. (**A**) Light production from a host *vqmR* transcriptional reporter (P*_vqmR_-luxCDABE*) following expression of the *tdh* genes under study in Δ*tdh V. cholerae*. (**B**) Light production from a phage *qtip* transcriptional reporter (P*_qtip_-luxCDABE*) following expression of the *vqmAφ* genes under study in Δ*tdh V. parahaemolyticus* in the absence and presence of DPO. (**C**) Light production from P*_qtip_-luxCDABE* following expression of *vqmAφ* genes in their respective host species (*V. parahaemolyticus*, *V. vulnificus*, or *S. enterica*). (**D**) Light production from P*_vqmR_-luxCDABE* following expression of the *vqmAφ* genes under study in Δ*tdh* Δ*vqmA V. cholerae*. (**A–D**) All experiments were performed in biological triplicate (*n* = 3). EV denotes the empty vector control. Symbols represent individual replicate values. Bars represent means. Error bars represent standard deviations. Cultures were treated with water (Uninduced) or arabinose and theophylline (Induced). DPO was added as noted. For statistical analyses, all treatments were compared within each sample. Significance was determined by two-way ANOVA with (**A, C, and D**) Šídák’s or (**B**) Tukey’s multiple comparisons test to determine adjusted *P* values: (**A**) ns = non-significant, ****P < 0.0001, (**B**) ns = non-significant, *P = 0.0254, ***P = 0.0003, and ****P < 0.0001, (**C**) ns = non-significant, *P = 0.0358, and ****P < 0.0001, and (**D**) ns = non-significant, ****P < 0.0001.

Our first goal was to assess the capacity of the strains carrying VP882-like phages to produce DPO. To do this, we compared the amino acid sequences of the 14 identified Tdh proteins. Using a 100% amino acid identity cutoff, we revealed six Tdh isoforms that cluster by species (Tdh^Vp^, Tdh^Vv^, Tdh^Vc^, Tdh^Sa^, Tdh^Se1^, and Tdh^Se2^; Fig. S3A). To assess whether the different Tdh isoforms are functional, we introduced each Tdh isoform into a Δ*tdh V. cholerae* DPO biosensor strain that is incapable of DPO production and that carries a transcriptional luciferase reporter for *vqmR* (P*_vqmR_-luxCDABE*), the downstream target of the DPO-VqmA complex (Fig. 4A). Activation of P*_vqmR_-luxCDABE* transcription tracks with DPO production (3, 4, 21). We ensured that all Tdh isoforms were produced using western blot. Equivalent amounts of the Tdh isoforms were made in *E. coli*, while in the Δ*tdh V. cholerae* DPO biosensor strain, some variability in abundance occurred (Fig. S4A and B). Despite these differences, all six Tdh isoforms produced DPO that activated VqmA to drive luciferase production in the *V. cholerae* biosensor strain to within two-fold of that driven by our reference Tdh (Tdh^C6706^) (Fig. 4A). These data show that each Tdh-harboring host strain has the capacity to produce DPO. To verify that the Tdh isoforms are active in their native hosts, we measured endogenous DPO production in cultures of *V. parahaemolyticus* RIMD 2210633, *V. vulnificus* ATCC 29306, *V. cholerae* C6706, *S. algae* ATCC 51192, and *S. enterica* serovar Typhimurium 14028s. The Tdh proteins in all of these strains are identical to the corresponding Tdh isoforms present in the VP882-like phage lysogenic hosts (Fig. S5A). Cell-free culture fluids from all tested strains activated *vqmR* transcription to levels significantly higher than did cell-free culture fluids from Δ*tdh* control strains. Thus, all strains under study produced DPO under our experimental conditions (Fig. S5B through D). Taken together, our results indicate that each VP882-like phage host species harboring a *tdh* gene can produce DPO.

### Most VP882-like phages encode a functional VqmAφ-Qtip module

Of the 15 VP882-like phages with associated host genomes, 12 carry an intact *vqmAφ* comprising six isoforms (VqmAφ^1–6^; Fig. 3A through C; Fig. S2A). To test whether these VqmAφ isoforms can detect DPO and, in response, activate transcription of the φVP882 antirepressor *qtip*, we introduced each VqmAφ isoform into our Δ*tdh V. parahaemolyticus* strain that does not produce DPO and that carries a transcriptional reporter for *qtip* (P*_qtip_-luxCDABE*; Fig. 4B). Western blot showed that all VqmAφ isoforms were produced in equivalent amounts in *E. coli* (Fig. S4C). However, despite multiple attempts using different antibodies, VqmAφ failed to react with imaging reagents following western blot of *V. parahaemolyticus* preparations. Nonetheless, in *V. parahaemolyticus*, both apo- and DPO-bound VqmAφ (holo-VqmAφ) activated P*_qtip_-luxCDABE* expression, with holo-VqmAφ being more active than apo-VqmAφ, demonstrating that the proteins were present. Specifically, VqmAφ^1^, VqmAφ^2^, VqmAφ^3^, and VqmAφ^5^ activated P*_qtip_-luxCDABE* expression in their apo-states, and in each case, P*_qtip_-luxCDABE* expression increased following DPO supplementation (Fig. 4B). The VqmAφ^1^, VqmAφ^2^, VqmAφ^3^, and VqmAφ^5^ isoforms are present across 9 of the 12 VP882-like phages with intact *vqmAφ-qtip* modules (Fig. 3A and C) and are distributed across *V. parahaemolyticus* (VqmAφ^1,2,3,5^), *V. vulnificus* (VqmAφ^3^), *S. enterica* (VqmAφ^3^), and *S. algae* (VqmAφ^3^) (Fig. 3C; Fig. S2A). VqmAφ^6^ (*Rikenellaceae*) activated P*_qtip_-luxCDABE* transcription in the apo-state but did not respond to DPO supplementation (Fig. 4B). This result is consistent with VqmAφ^6^ possessing an eight amino acid deletion in the DPO-binding PAS domain (Fig. 3B; Fig. S2A) and the fact that *Rikenellaceae* MAG302 does not harbor an identifiable *tdh* gene (Fig. 3C; Fig. S3A). VqmAφ^4^ (*S. enterica*) failed to activate P*_qtip_-luxCDABE* expression in our *V. parahaemolyticus* reporter system (Fig. 4B). We presume, based on >95% amino acid identity between the VqmAφ isoforms, that the observed variations are due to differences in protein activity rather than differences in protein abundance. Collectively, our data demonstrate that 10 of the 12 VP882-like phages associated with a host strain and with an intact *vqmAφ-qtip* module encode a VqmAφ protein that can activate the lysis-inducing *qtip* gene, and 9 of these VqmAφ proteins do so in response to DPO.

We also investigated whether the VqmAφ isoforms are functional in their native host species. Because we do not have access to the native host strains of the VP882-like phages, we produced each VqmAφ isoform in a strain that was a species match to its native host: *V. parahaemolyticus* RIMD 2210633 (VqmAφ^1,2,3,5^), *V. vulnificus* ATCC 29306 (VqmAφ^3^), and *S. enterica* 14028s (VqmAφ^3,4^). Additionally, each strain was engineered to carry a P*_qtip_-luxCDABE* transcriptional reporter, and they all naturally produce their own DPO (Fig. S5C). *S. algae* is incompatible with our reporter system, and we do not have access to any *Rikenellaceae* family isolates. Thus, we could not test VqmAφ^3^ or VqmAφ^6^ in those two hosts. In every tested case, production of the VqmAφ isoform in its endogenous host species drove a significant increase in P*_qtip_-luxCDABE* transcription (Fig. 4C). The isoform VqmAφ^3^ activated comparable levels of P*_qtip_-luxCDABE* transcription in three different species, indicating no restriction to a single host species. The *S. enterica-*specific VqmAφ^4^ isoform did not activate P*_qtip_-luxCDABE* transcription in the heterologous *V. parahaemolyticus* host (Fig. 4B) but did drive increased P*_qtip_-luxCDABE* transcription in the *S. enterica* host (Fig. 4C). Nonetheless, the VqmAφ^4^ isoform was 100-fold less active than the VqmAφ^3^ isoform in the *S. enterica* host (Fig. 4C). The polymorphisms in the VqmAφ^4^ isoform may be less detrimental in *S. enterica* than in *V. parahaemolyticus*; still, the data show that the VqmAφ^4^ isoform appears to generally be a poor activator of P*_qtip_-luxCDABE* transcription.

### All VqmAφ isoforms activate transcription of the vibrio-specific host *vqmR* gene

In *V. parahaemolyticus* and *V. cholerae*, beyond activating the phage *qtip* gene, VqmAφ from φVP882 (VqmAφ^1^) can also regulate host transcription by binding to and activating the *vqmR* promoter (2, 9). Due to the restriction of the VqmAR host quorum-sensing system to vibrios, we aimed to determine whether the six VqmAφ isoforms, including those not harbored by a vibrio host, can activate *vqmR* transcription or if some isoforms have evolved away from this regulatory relationship. To this end, we produced each VqmAφ isoform in the Δ*tdh* Δ*vqmA V. cholerae* strain that neither produces nor detects DPO (Fig. 4D; Fig. S4D). The strain also carries a transcriptional reporter for *vqmR* (P*_vqmR_-luxCDABE*). All six VqmAφ isoforms activated P*_vqmR_-luxCDABE* transcription (Fig. 4D). VqmAφ^1^, VqmAφ^2^, VqmAφ^3^, and VqmAφ^6^ isoforms were produced at equivalent levels, while VqmAφ^4^ and VqmAφ^5^ were produced at similar but lower levels, which may account for their corresponding lower levels of activation of *vqmR* transcription (Fig. 4D; Fig. S4D).

## DISCUSSION

We discovered that φVP882 is a member of a taxon of VP882-like phages that infect diverse bacterial species. All but one of these bacterial hosts encode a functional Tdh enzyme, and thus, likely produce DPO. We demonstrated that the majority of VP882-like phages harbor a VqmAφ capable of detecting DPO and, in response, driving *qtip* transcription. Each VqmAφ isoform was also functional when expressed in laboratory strains of the native species in which it exists as a lysogen. Finally, regarding influencing host behavior, every VqmAφ isoform under study could activate transcription from the vibrio host *vqmR* promoter.

We identified 22 highly similar VP882-like phages across at least six host species: *V. parahaemolyticus*, *V. vulnificus*, *V. cholerae*, *S. algae*, *S. enterica*, and a *Rikenellaceae* family bacterium (Table S1). Our unbiased searching strategy delivered sequences primarily from NCBI. Thus, the associated host strains for the extracted phages were largely biased toward common nosocomial pathogens including *K. pneumoniae*, *E. coli*, and *S. enterica* (Supplementary Data set 1). This outcome is likely due to the public health focus that biases entries in NCBI databases (22, 23). Specifically, as of November 2025, there are ~2.8 million bacterial genomes in RefSeq (24), and *K. pneumoniae*, *E. coli*, and *S. enterica* make up 38% of these genomes. With respect to our findings, while 4 VP882-like phages are harbored by *S. enterica* strains, the other 11 VP882-like phages with associated hosts are in *Vibrio*, *Shewanella*, and *Rikenellaceae* bacteria, all of which are underrepresented in RefSeq (22). The remaining seven VP882-like phages that were extracted from metagenomic data within IMG/VR were isolated in asphalt lakes, marine sediment, and industrial wastewater (Table S1). Collectively, our data indicate that VP882-like phages predominantly occur in underrepresented pathogens and environmental bacteria and not in public-health-associated bacterial pathogens and patient samples. With increased environmental sequencing efforts coupled with methodological advancement (25–28), we anticipate the discovery of additional VP882-like phages.

Our data suggest that VP882-like phages across a variety of host species maintain functionality of the *vqmAφ-qtip* module and have the capacity to monitor host cell density via tracking of the surrounding DPO concentration. Whether other host or phage components are involved in regulation of the *vqmAφ-qtip* modules remains to be investigated. VqmAφ and VqmA are unlike other quorum-sensing receptors in that VqmAφ and VqmA can activate their target promoters in the absence of their partner autoinducer ligand (3, 4, 9). High levels of apo-VqmAφ are sufficient to drive *qtip* transcription and lytic induction in a φVP882 lysogen that lacks the ability to produce its own DPO (Δ*tdh*) (9). We presume that, in natural lysogenic contexts, VqmAφ protein levels must be tightly controlled to avoid premature lytic induction, that is, lysis when potential new hosts are sparse. Our experiments do not address whether host species-specific factors or individual phage-specific factors exist and regulate VqmAφ production or activity.

All VqmAφ isoforms studied here except VqmAφ^4^ could activate *qtip* transcription. All VqmAφ isoforms, including VqmAφ^4^, could activate transcription of the gene encoding the vibrio host VqmR sRNA. VqmAφ^4^ differs from VqmAφ^3^, one of the strongest activators of both *qtip* and *vqmR* transcription, at two amino acid residues—D137E and A175D. We previously demonstrated that a mutation in VqmAφ from φVP882 (here denoted VqmAφ^1^) at residue 176 (K176Q) significantly reduced the ability of VqmAφ^1^ to bind the *qtip* promoter while having no effect on binding to the *vqmR* promoter (9). We hypothesize that harboring an Asp residue at position 175 prevents VqmAφ^4^ from binding to the *qtip* promoter. This polymorphism may represent an early step in phage domestication. Lysogeny by temperate phages frequently delivers benefits to the host. Thus, degradation of phage genomes is a mechanism that can lock in benefits of lysogeny while eliminating risks to the host (29, 30). Abolishing VqmAφ-triggered lysis via elimination of binding at *qtip* while maintaining the ability of VqmAφ to regulate host genes could remove the danger of density-dependent lysis while preserving benefits of density-dependent VqmAφ-driven transcription of host genes.

Since the discovery and characterization of φVP882 as the first temperate phage to integrate host quorum-sensing cues into its lysis-lysogeny decision-making, other quorum-sensing-responsive bacteriophages, including phages that both produce and detect autoinducers, have been discovered (2, 6, 31). Our results demonstrate that VP882-like phages, and linear plasmid phages in general, are widely dispersed in nature. Further study of VP882-like phages in vibrio and non-vibrio hosts could identify general and species-specific mechanisms of phage-host communication. More broadly, studies of linear plasmid phages have yielded new insights into phage-bacterial chemical communication, smORF-mediated phage-phage competition during polylysogeny, and phage-dependent inter-bacterial competition (2, 6, 10). By mining expanding viral sequence databases for linear plasmid phages, new biological phenomena that deepen our understanding of phage biology, inter-domain chemical communication, and inter-phage and inter-bacterial competitive dynamics could be uncovered.

## MATERIALS AND METHODS

### Viral taxonomy and genomic analysis

Linear plasmid phages were identified using a strategy similar to reference (6). Searches were performed across NCBI nt, IMG/VR v4 (13), CHVD v1.1 (14), GOV2 (12), GPD (15), and MGV (16). All sequences were downloaded in April 2025.

For NCBI nt, TelN proteins were identified by blastp with the TelN protein sequence from three linear plasmid phages (φVP882: YP_001039865.1, φ63: WP_372435025.1, and φ72: AKN37353.1) (6, 11) against the NCBI nr database. Hits were filtered for >40% identity and >80% query coverage (*n* = 1,785). Protein IDs were used to extract phage genomes from NCBI nt. The Identical Protein Groups were gathered for each protein ID and all GenBank contig accession IDs were extracted. These nucleotide accessions were used to fetch 16,409 contigs (length > 15 kb) and their annotated proteins. Phage status was verified by assessing the collected contigs using VIBRANT (v1.2.1) (32). Sequences called as phages were used in further analyses.

For IMG/VR, CHVD, GOV2, GPD, and MGV, database files were annotated with VIBRANT (32) to obtain predicted protein fasta files. TelN proteins were identified by profile HMM search with HMMER3 (v.3.3.2) (33) for domain PF16684 (Pfam v.37) (34) against VIBRANT-generated protein databases.

The acquired *telN*-encoding phage genomes from all six databases were dereplicated with cd-hit-est (35) (parameters: “-c 0.99 -aS 1.0g 1 -d 0”). All associated protein sequences were deposited into a single combined database and submitted to vConTACT2 (v.0.11.3) (36) with the Prokaryotic Viral RefSeq database (v.211) (37) to determine linear plasmid phage taxonomy. GenBank files were extracted for all genomes in viral clusters of interest. Two additional VP882-like phages, uncovered by blastp against NCBI nr, were added post-analysis, as their contigs were below the length filter of the analysis pipeline. Whole-genome synteny was calculated and visualized with clinker (v.0.0.31) (38). Nucleotide and protein alignments were performed in Geneious Prime (v.2025.0.3) using MUSCLE (39). Heatmaps were generated in R with pheatmap (v.1.0.13) using % identities calculated in Geneious Prime.

### Bacterial strains, reagents, and growth conditions

Bacterial strains and plasmids used in this study are listed in Table S2. Overnight cultures were grown with aeration in Lysogeny Broth (LB-Miller, BD-Difco) or on plates containing LB +1.5% agar at 37°C (*V. cholerae* and *S. enterica*) or in LB with 2% NaCl (LM) or on plates containing LM +1.5% agar at 30°C (*V. parahaemolyticus*, *V. vulnificus*, and *S. algae*). Unless stated otherwise, all experiments were performed in M9 medium with 0.5% glucose, 0.4% casamino acids, and 200 mM NaCl (M9-gluc-CAA-HS). Antibiotics were used at: 50 µg mL^−1^ kanamycin (Kan, GoldBio) and 10 µg mL^−1^ chloramphenicol (Cm, Sigma). l-arabinose (Sigma), theophylline (Sigma), l-threonine (Sigma), and DPO (Aablocks, #60187-00-0) were used as indicated.

### Strain and plasmid construction

All primers and synthetic DNA fragments used for plasmid construction, listed in Table S3, were obtained from Integrated DNA Technologies. FastCloning was employed for plasmid assembly (40). Briefly, PCR with Phusion (NEB) or iProof (Bio-Rad) polymerase was used to generate cloning inserts and linear plasmid backbone DNA. In cases in which inserts could not be generated by PCR, fragments were synthesized by Integrated DNA Technologies. Plasmid backbone DNA was treated with DpnI (NEB) to remove PCR template DNA. Cloning inserts and linear plasmid backbones were added to chemically competent *E. coli* cells, and plasmid assembly was carried out by the transformed cells. All assembled plasmids were verified by sequencing. Transfer of plasmids into *V. parahaemolyticus*, *V. cholerae*, *V. vulnificus*, *S. algae*, and *S. enterica* strains was accomplished by conjugation with a conjugal helper strain as described (41) or a conjugative DAP-auxotroph as described (42) (for *S. enterica*). Conjugation was followed by selective plating on LM or LB plates supplemented with appropriate antibiotics.

### Bioassay for quantitation of DPO production

When *tdh* was provided by complementation: Cultures of the *V. cholerae* DPO biosensor (Δ*tdh* Δ*lacZ*::P*_vqmR_-luxCDABE*) carrying arabinose/theophylline-inducible *tdh* alleles (pXBCm-P*_bad-riboswitch_-tdh^X^-FLAG*) were diluted 1:1,000 in M9-gluc-CAA-HS supplemented with 10 mM l-threonine and appropriate antibiotics. Tdh production was induced with 0.6% l-arabinose and 200 µM theophylline. Diluted cultures were dispensed (200 µL) into white-wall/clear-bottom 96-well plates (Corning Costar). Optical density and bioluminescence were measured every 5 min with a BioTek Synergy Neo2 Multi-Mode plate reader.

When DPO was added exogenously: The conditions below were chosen to optimize growth for each strain while keeping as many variables constant as possible. Three independent colonies were picked from overnight agar plates for each putative DPO-producing strain. Colonies were inoculated into M9 medium supplemented with either 0.5% glycerol, 0.4% casamino acids, and 340 mM NaCl (*V. parahaemolyticus*, *V. cholerae*, and *V. vulnificus*), 0.5% glucose, 0.4% casamino acids, and 340 mM NaCl (*S. algae*), or 0.5% glucose and 0.4% casamino acids (*S. enterica*). All media were supplemented with 10 mM l-threonine, as it is the precursor for DPO production (3). Cultures were grown with aeration at 30°C (*V. parahaemolyticus*, *V. vulnificus*, and *S. algae*) or 37°C (*V. cholerae* and *S. enterica*) for 16 h. Culture fluids were collected and filtered through a 96-well 0.22 µm filter plate (MilliporeSigma). The *V. cholerae* DPO biosensor (Δ*tdh* Δ*vqmAR* Δ*lacZ*::P*_vqmR_-luxCDABE* Δ*vc1807*::P*_bad_-vqmA*) was diluted 1:1,000 in M9-gluc-CAA-HS supplemented with 0.01% l-arabinose to induce low-level VqmA production, which shows the optimal dynamic range. Diluted cultures were dispensed (150 µL) into white-wall/clear-bottom 96-well plates (Corning Costar) and treated with 25% cell-free culture fluid or sterile medium (50 µL). Separate wells of diluted biosensor culture were treated with known amounts of DPO consisting of five-fold dilutions from 1 mM to 12.8 nM to generate a standard curve. Optical density and bioluminescence were measured every 5 min with a BioTek Synergy Neo2 Multi-Mode plate reader to calculate relative light units (Bioluminescence/OD_600_). DPO concentrations in the collected cell-free culture fluids were interpolated by plotting experimental Bioluminescence/OD_600_ values against the DPO standard curve.

### Bioassay for quantitation of *qtip* induction

For heterologous expression: Cultures of Δ*tdh V. parahaemolyticus* carrying the P*_qtip_-luxCDABE* reporter and arabinose/theophylline-inducible *vqmAφ* alleles (pXBCm-P*_bad-riboswitch_-vqmAφ^X^-FLAG*) were diluted 1:1,000 in M9-gluc-CAA-HS. VqmAφ production was induced with 0.6% arabinose and 200 µM theophylline. 10 µM DPO or water (vehicle) was administered to uninduced and induced samples.

For endogenous host species expression: Cultures of *V. parahaemolyticus*, *V. vulnificus*, and *S. enterica* carrying the P*_qtip_-luxCDABE* reporter and arabinose/theophylline-inducible *vqmAφ* alleles (pXBCm-P*_bad-riboswitch_-vqmAφ^X^-FLAG*) were diluted 1:1,000 in LM (*V. parahaemolyticus* and *V. vulnificus*) or LB (*S. enterica*). VqmAφ production was induced with 0.6% arabinose and 200 µM theophylline.

In all cases, diluted cultures were dispensed (200 µL) into white-wall/clear-bottom 96-well plates (Corning Costar). Optical density and bioluminescence were measured every 5 min with a BioTek Synergy Neo2 Multi-Mode plate reader.

### Western blot analysis

Expression in *E. coli:* Overnight cultures of strains harboring C-terminal FLAG-tagged arabinose/theophylline-inducible *tdh* alleles (pXBCm-P*_bad-riboswitch_-tdh^X^-FLAG*), C-terminal FLAG-tagged arabinose/theophylline-inducible *vqmAφ* alleles (pXBCm-P*_bad-riboswitch_-vqmAφ^X^-FLAG*), or empty vector controls were diluted 1:1,000 in LB medium supplemented with 0.6% l-arabinose and 200 µM theophylline and grown with aeration at 37°C for 16 h. Cells were collected by centrifugation at 13,000 rpm for 2 min, pellets were resuspended in BugBuster Protein Extraction Reagent (MilliporeSigma) supplemented with 50 µg mL^−1^ lysozyme and 25 U mL^−1^ benzonase nuclease, and the cells were allowed to lyse at room temperature for 30 min. Fluorescent Compatible Sample Buffer (Invitrogen) was added, bringing each sample to a final concentration of 0.2 OD_600_ μL^−1^.

Expression in *V. cholerae*: Overnight cultures of strains harboring C-terminal FLAG-tagged arabinose/theophylline-inducible *tdh* alleles (pXBCm-P*_bad-riboswitch_-tdh^X^-FLAG*), C-terminal FLAG-tagged arabinose/theophylline-inducible *vqmAφ* alleles (pXBCm-P*_bad-riboswitch_-vqmAφ^X^-FLAG*), or empty vector controls were diluted 1:100 in M9-gluc-CAA-HS supplemented with 0.6% l-arabinose, 200 µM theophylline, and in the case of Tdh constructs, 10 mM l-threonine and grown with aeration at 37°C to OD_600_ ~0.8. Cells were collected by centrifugation at 13,000 rpm for 2 min, pellets were resuspended in BugBuster Protein Extraction Reagent (MilliporeSigma) supplemented with 50 µg mL^−1^ lysozyme and 25 U mL^−1^ benzonase nuclease, and the cells were allowed to lyse at room temperature for 30 min. Fluorescent Compatible Sample Buffer (Invitrogen) was added, bringing each sample to a final concentration of 0.08 OD_600_ μL^−1^.

In all cases, following denaturation for 30 min at 98°C, 40 µL of each sample was separated on a 12% Mini-PROTEAN TGX Stain-Free gel (Bio-Rad). Stain-free gels were activated by exposure to UV light for 1.5 min. Proteins were transferred from the gels to Low-Fluorescence PVDF membranes (Invitrogen). Membranes were blocked for 1 h in PBST (PBS supplemented with 0.03% Tween) with 5% milk. Subsequently, membranes were incubated for 1 h with a monoclonal αFLAG-peroxidase antibody (Millipore Sigma, #A8592) at a 1:5,000 dilution in PBST with 5% milk. After washing four times with PBST for 10 min each, membranes were exposed using the SuperSignal West Femto Maximum Sensitivity Substrate (ThermoFisher) or the SuperSignal West Atto Ultimate Sensitivity Substrate (ThermoFisher) (for VqmAφ production in *V. cholerae*). Target proteins and total protein were visualized with an Amersham ImageQuant 800 biomolecular imager.

## Data Availability

All data sets used in this study are publicly available. Custom Python scripts used in this study are available upon request.

## SUPPLEMENTAL MATERIAL

Data set S1 mbio.02320-25-s0001.xlsx Identifiers for all genomes used in this study.

Data set S2 mbio.02320-25-s0002.xlsx vConTACT-assigned viral clusters for all phage genomes.

Supplemental Information mbio.02320-25-s0003.pdf Supplemental figures and tables.

ASM does not own the copyrights to Supplemental Material that may be linked to, or accessed through, an article. The authors have granted ASM a non-exclusive, world-wide license to publish the Supplemental Material files. Please contact the corresponding author directly for reuse.
