# Supplementary material for: A family of linear plasmid phages that detect a quorum-sensing autoinducer exists in multiple bacterial species
Source: mBio. 2025 Dec 12;17(1):e02320-25. doi: 10.1128/mbio.02320-25 (PMC12802170; doi:10.1128/mbio.02320-25)
Supplement: Supplemental Information — Supplemental figures and tables. [file mbio.02320-25-s0003.pdf]

SUPPLEMENTARY MATERIAL

A family of linear plasmid phages that detect a quorum-sensing autoinducer exists in multiple bacterial species.

Santoriello, FJ & Bassler, BL

Figure S1. Hapunaviruses are subdivided into VP882-like phages and HAP-1-like phages.

Figure S2. VqmAφ and cI vary across VP882-like phages, whereas Qtip is highly conserved.

Figure S3. LuxO-OpaR(LuxR, HapR) and VqmAR quorum-sensing proteins are restricted to vibrios, while the autoinducer synthases Tdh and LuxS are conserved across genera.

Figure S4. Abundances of Tdh and VqmAφ isoforms examined by western blot.

Figure S5. Tdh isoforms encoded by the strains under study produce DPO.

Table S1. Genome metadata for VP882-like linear plasmid phages.

Table S2. Strains and plasmids used in this study.

Table S3. Primers and synthetic DNA fragments used in this study.

Supplementary Dataset 1. Identifiers for all genomes used in this study.

Supplementary Dataset 2. vConTACT assigned viral clusters for all phage genomes.

Supplemental References

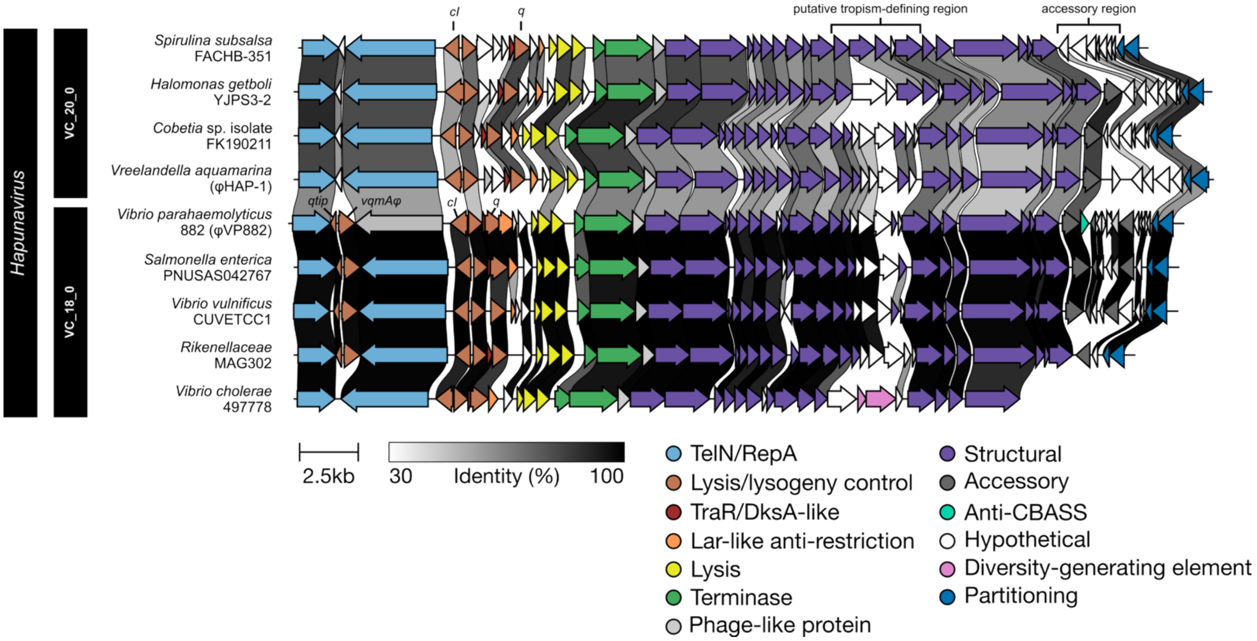

Fig S1 Hapunaviruses are subdivided into VP882-like phages and HAP-1-like phages. Genome synteny of HAP-1-like (VC_20_0) and VP882-like (VC_18_0) linear plasmid phages. Host species and strain are provided on the left. Arrows represent genes colored according to their annotated functions. Gene homologs in neighboring sequences are connected by shaded links. Shading represents the % identity between the amino acid sequences of the proteins encoded by the homologous genes. The absence of a link indicates less than 30% amino acid identity between proteins encoded by neighboring genes or the absence of a homolog in the neighbor.

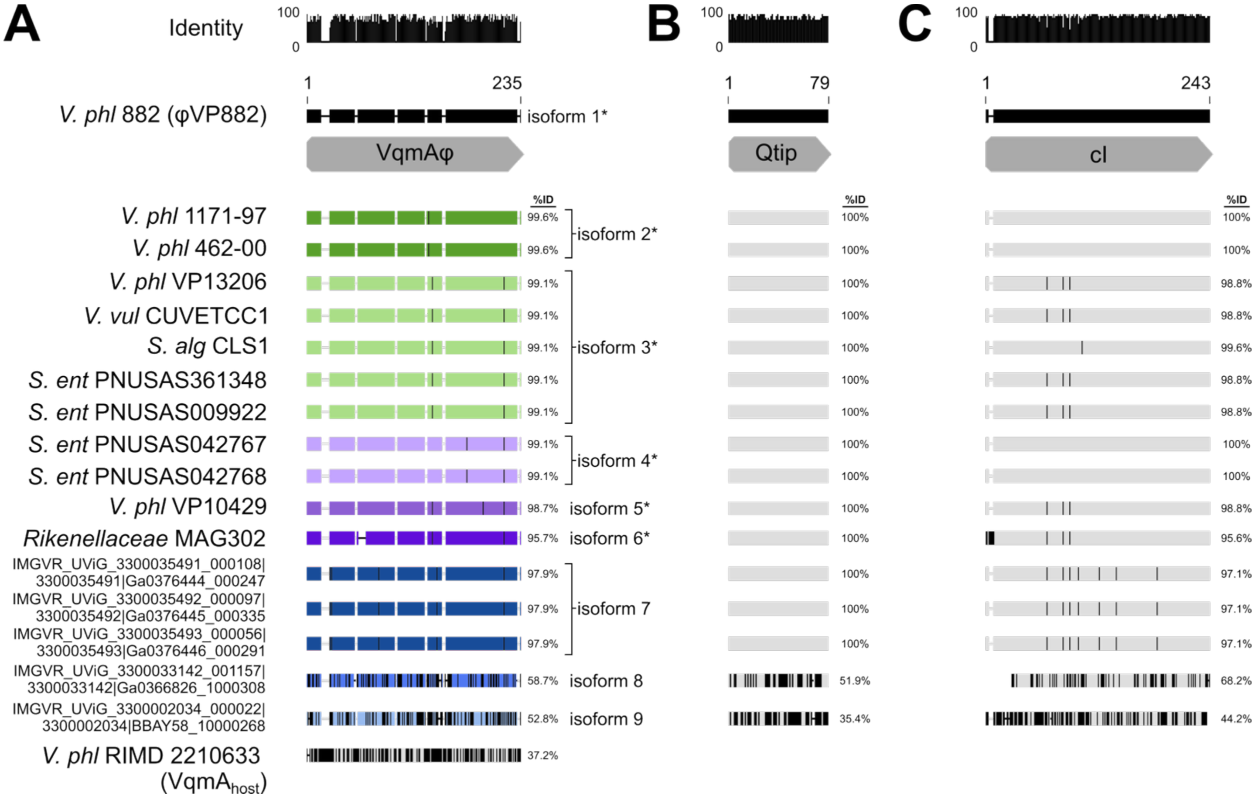

Fig S2 VqmAφ and cI vary across VP882-like phages, whereas Qtip is highly conserved. Pairwise amino acid alignments of (A) VqmAφ, (B) Qtip, and (C) cI proteins from the 17 VP882-like phages with intact *vqmAφ-qtip* modules (see Fig. 3B of the main text). Host species and strain are provided on the left (*V. phl* = *Vibrio parahaemolyticus*, *V. vul* = *Vibrio vulnificus*, *V. cho* = *Vibrio cholerae*, *S. ent* = *Salmonella enterica*, *S. alg* = *Shewanella algeae*). Sequences designated IMGVR were collected from metagenomic data and thus, do not have associated host strains. Horizontal bars represent the homologous protein sequences, and black vertical lines within the bars represent amino acid differences relative to the VqmAφ, Qtip, and cI proteins from φVP882. Thin black horizontal lines denote gaps in sequences compared to the reference sequence. Thin gray horizontal lines denote gaps in sequences compared to any sequence other than the reference sequence. In (A), VqmAφ proteins are colored and labeled by isoform. Isoforms marked with asterisks were used in assays in the main text. The $VqmA_{host}$ protein sequence from *V. parahaemolyticus* RIMD 2210633 was included to demonstrate that while VqmAφ isoforms 8 and 9 vary most among VqmAφ homologs, they share greater identity with VqmAφ than the identity shared between $VqmA_{host}$ and VqmAφ. This finding supports the logic that all the VqmAφ isoforms shown should be considered VqmAφ rather than some other transcription factor.

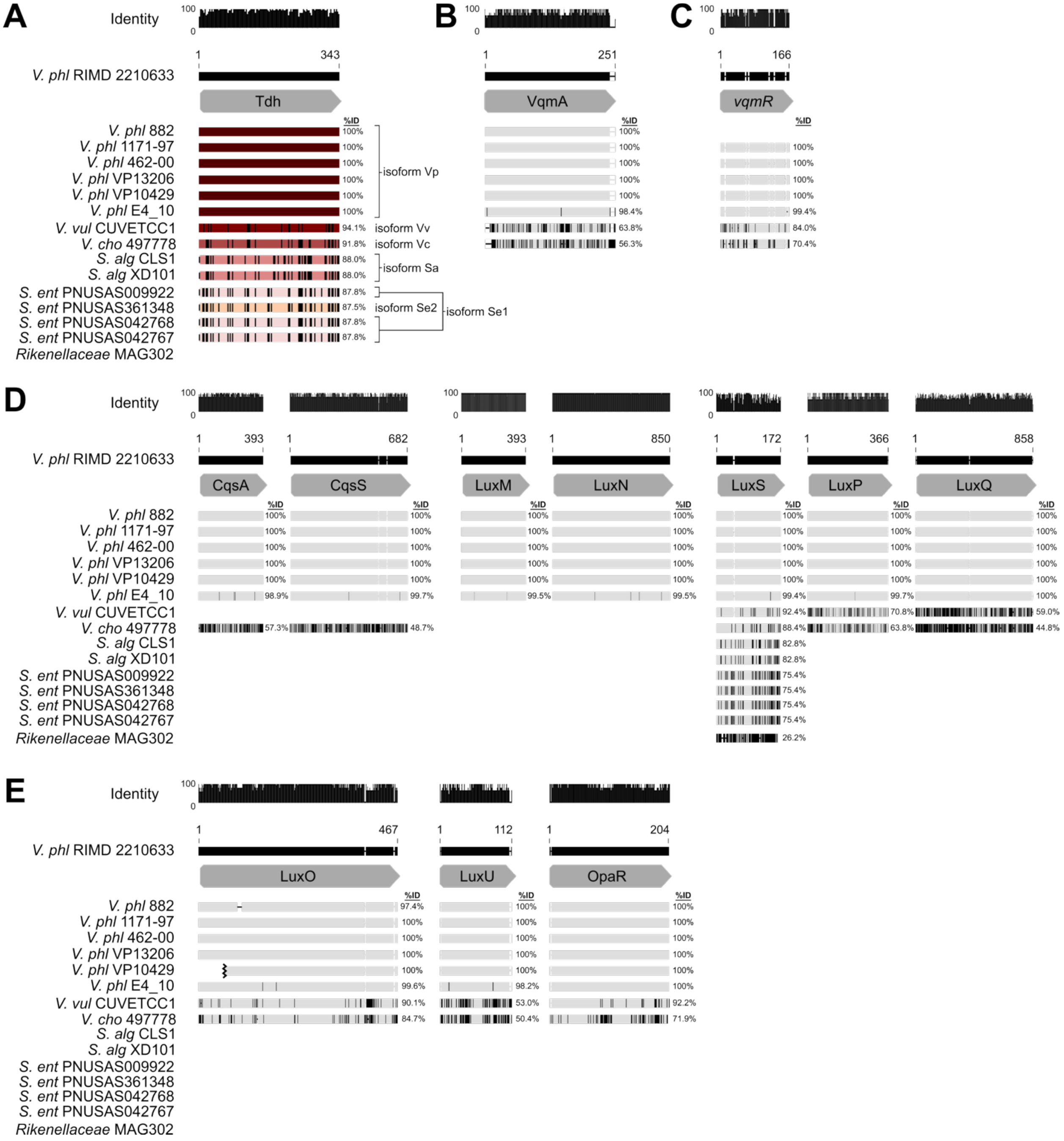

Fig S3 LuxO-OpaR(LuxR, HapR) and VqmAR quorum-sensing proteins are restricted to vibrios, while the autoinducer synthases Tdh and LuxS are conserved across genera. Pairwise alignments of (A) Tdh, (B) VqmA$_{host}$, (C) *vqmR*, (D) the autoinducer synthase and receptor proteins associated with the LuxO-OpaR(LuxR, HapR) pathway, and (E) LuxO, LuxU, and OpaR(LuxR, HapR) from 15 host strains harboring VP882-like phages (see Fig. 3A,C of the main text). (A-E) Host species and strain are provided on the left (*V. phl* = *Vibrio parahaemolyticus*, *V. vul* = *Vibrio vulnificus*, *V. cho* = *Vibrio cholerae*, *S. ent* = *Salmonella enterica*, *S. alg* = *Shewanella algeae*). Horizontal bars represent the homologous (A,B,D,E) protein or (C) nucleotide sequences, and black vertical lines within the bars represent (A,B,D,E) amino acid or (C) nucleotide differences relative to the corresponding sequences from *V. parahaemolyticus* RIMD

2210633. Thin black horizontal lines denote gaps in sequences compared to the reference sequence. Thin gray horizontal lines denote gaps in sequences compared to any sequence other than the reference sequence. (A) Tdh proteins colored and labeled by isoform. (E) The black jagged mark in the *V. phl* VP10429 sequence denotes the end of the contig.

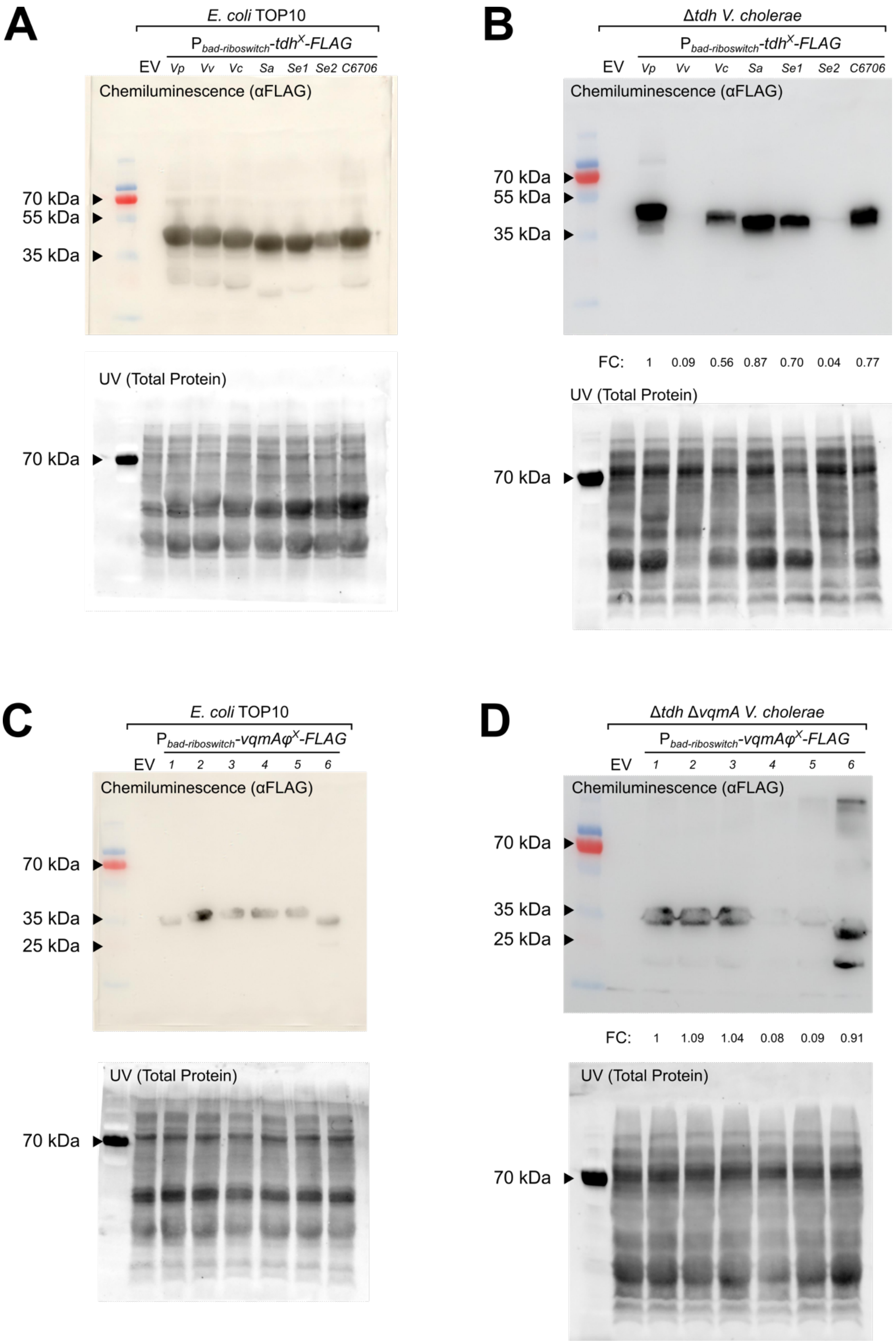

A
E. coli TOP10
Pbad-riboswitch-tdhX-FLAG
EV Vp Vv Vc Sa Se1 Se2 C6706
Chemiluminescence (αFLAG)
70 kDa
55 kDa
35 kDa
UV (Total Protein)
70 kDa
B
Δtdh V. cholerae
Pbad-riboswitch-tdhX-FLAG
EV Vp Vv Vc Sa Se1 Se2 C6706
Chemiluminescence (αFLAG)
70 kDa
55 kDa
35 kDa
FC: 1 0.09 0.56 0.87 0.70 0.04 0.77
UV (Total Protein)
70 kDa
C
E. coli TOP10
Pbad-riboswitch-vqmAφX-FLAG
EV 1 2 3 4 5 6
Chemiluminescence (αFLAG)
70 kDa
35 kDa
25 kDa
UV (Total Protein)
70 kDa
D
Δtdh ΔvqmA V. cholerae
Pbad-riboswitch-vqmAφX-FLAG
EV 1 2 3 4 5 6
Chemiluminescence (αFLAG)
70 kDa
35 kDa
25 kDa
FC: 1 1.09 1.04 0.08 0.09 0.91
UV (Total Protein)
70 kDa

Fig S4 Abundances of Tdh and VqmAφ isoforms examined by western blot. (A,B) Western blot of FLAG-tagged Tdh isoforms expressed in (A) *E. coli* and (B) Δ*tdh* Δ*lacZ*::P_{vqmR}-*luxCDABE V. cholerae,* used in Fig. 4A. (C,D) Western blot of FLAG-tagged VqmAφ isoforms expressed in (C) *E. coli* and (D) Δ*tdh* Δ*vqmA* Δ*lacZ*::P_{vqmR}-*luxCDABE V. cholerae*, used in Fig. 4D. (A-D) Blotting analyses were performed in duplicate, and one representative blot is shown. The top images are the western blots, and the bottom images are fluorophore-bound total transferred protein. Tdh and VqmAφ protein levels were normalized to total protein level. (B,D) Fold-change (FC) was calculated against Tdh^{Vp} for Tdh isoforms and against $VqmA\varphi^{1}$ for VqmAφ isoforms. (A,B) Expected molecular weight for Tdh isoforms is approximately 38 kDa. (C,D) Expected molecular weight for VqmAφ isoforms is approximately 27 kDa; apparently the protein runs at a slightly higher molecular weight.

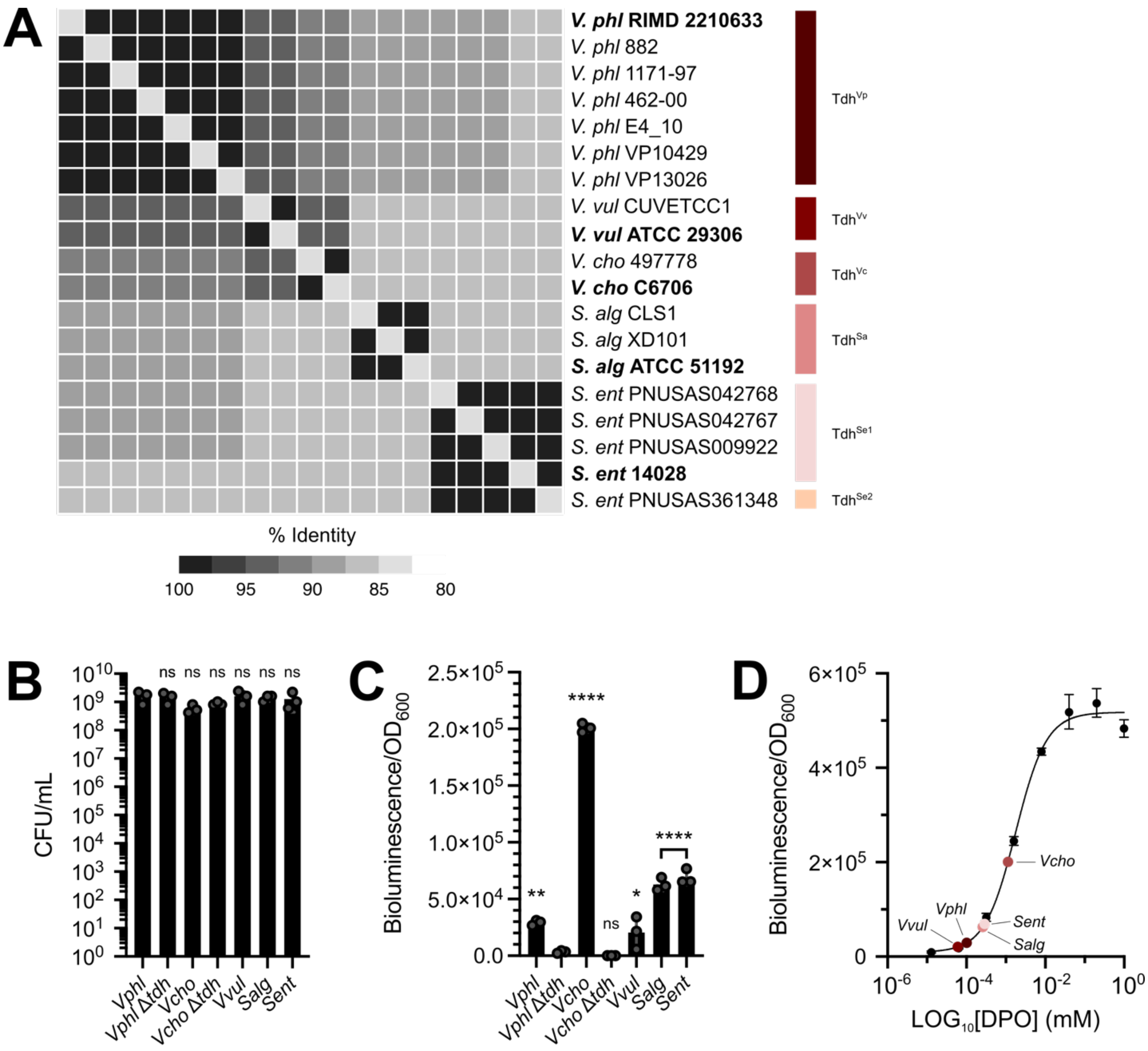

Fig S5 Tdh isoforms encoded by the strains under study produce DPO. (A) Pairwise heatmap of amino acid identities between the Tdh proteins encoded by VP882-like phage lysogenic hosts and corresponding laboratory isolates. Host species and strain are provided on the right (*V. phl* = *Vibrio parahaemolyticus*, *V. vul* = *Vibrio vulnificus*, *V. cho* = *Vibrio cholerae*, *S. alg* = *Shewanella algeae*, *S. ent* = *Salmonella enterica*). Laboratory isolates are bolded. Blocks corresponding to each Tdh isoform are indicated by colored bars to the right. (B) Colony forming units (CFUs) in cultures of the indicated strains from which cell-free culture fluids were collected. CFUs were measured at the time of culture fluid collection. (C) Light production from the host-encoded P_{vqmR}-*luxCDABE* transcriptional fusion following addition of cell-free culture fluids from the indicated strains to the Δ*tdh* Δ*vqmAR* Δ*lacZ*::P_{vqmR}-*luxCDABE* Δ*vc1807*::P_{bad}-*vqmA* *V. cholerae* DPO biosensor strain. Bioluminescence was normalized to OD_{600} of the biosensor strain. (D) Light production from the host-encoded P_{vqmR}-*luxCDABE* transcriptional fusion following the addition of DPO standards to the *V. cholerae* DPO biosensor strain. The standard curve was fit with a Four-Parameter Logistic curve (R-squared = 0.99). Normalized bioluminescence values from the tested cell-free culture fluids fit to the standard curve are shown with circles, the colors of which correspond to the Tdh isoforms in (A). (B-D) All experiments were performed in biological triplicate

(n = 3). Black circles represent individual replicate values. Error bars represent standard deviations. (B,C) Bars represent means. For statistical comparisons, all samples were compared to (B) *Vphl* or (C) *Vphl* ∆*tdh*. Significance was determined by one-way ANOVA with Dunnet's multiple comparisons test to determine adjusted p-values: (B) ns = non-significant, (C) ns = non-significant, *p = 0.0290, **p = 0.0014, **** p<0.0001.

Table S1. Genome metadata for VP882-like linear plasmid phages.

Host Strain [Accession; DB]	vConTACT2 Viral Cluster	Isolation Date	Isolation Location	Source
Vibrio parahaemolyticus 882 (1) [EF057797.1 ; GenBank]	VC_18_0	unknown (published 2009)	Taiwan	unknown
Vibrio parahaemolyticus 1171-97 [JAGJRA010000026.1 ; GenBank]	VC_18_0	1997	Peru: Moquegua	clinical
Vibrio parahaemolyticus 462-00 [JAGJPH010000018.1; GenBank]	VC_18_0	2000	Peru: Lima	clinical
**Vibrio parahaemolyticus* 461-00 [JAGJPI010000013.1; GenBank]	n/a	2000	Peru: Lima	clinical
***Vibrio parahaemolyticus* E4_10 [NNHH01000049-NNHH01000051; GenBank]	n/a	2014	China: Zhejiang	fish
Vibrio parahaemolyticus str VP13026 [DAHSFQ010000024.1; GenBank]	VC_18_0	2013	China: Shenzhen	clinical
Vibrio parahaemolyticus str VP10429 [DAHSQU010000027.1; GenBank]	VC_18_0	2010	China: Shenzhen	clinical
Vibrio vulnificus str CUVETCC1 [JALGBD010000034.1; GenBank]	VC_18_0	2021	Thailand: Chachoengsao	fish
Vibrio cholerae str 497778 [AAYQKK010000034.1; GenBank]	VC_18_0	2017	unknown	clinical
Salmonella enterica str PNUSAS042767 [AAGBFI010000022.1; GenBank]	VC_18_0	2018	USA	clinical
Salmonella enterica str PNUSAS042768 [AAFWQT010000024.1; GenBank]	VC_18_0	2018	USA	clinical
Salmonella enterica str PNUSAS361348 [ABNQCN010000017.1; GenBank]	VC_18_0	2023	USA	clinical
Salmonella enterica str PNUSAS009922 [AAEKWQ010000030.1; GenBank]	VC_18_0	2017	USA	clinical
Shewanella algae str XD101 [JBDIOS010000019.1; GenBank]	VC_18_0	2023	China: Sanya	seawater
***Shewanella algae* str CLS1 [LTBI01000116, LTBI01000117, LTBI01000119, LTBI01000120, LTBI01000131, LTBI01000132, LTBI01000137, LTBI01000141, LTBI01000147, LTBI01000150, LTBI01000152; GenBank]	n/a	2014	Taiwan	clinical

Rikenellaceae bacterium MAG302 [JBCQTA010000021.1; GenBank]	VC_18_0	2017	New Zealand: Little Barrier Island, Auckland	fish gut metagenome
[IMGVR_UViG_3300011259_000019|3300011259|Ga0151662_1001257; IMG/VR]	VC_18_0	2015	Japan: Japan Sea near Toyama Prefecture	marine sediment
[IMGVR_UViG_3300035491_000108|3300035491|Ga0376444_000247; IMG/VR]	VC_18_0	2007	Trinidad and Tobago: La Brea, Pitch Lake	asphalt lake
[IMGVR_UViG_3300035492_000097|3300035492|Ga0376445_000335; IMG/VR]	VC_18_0	2007	Trinidad and Tobago: La Brea, Pitch Lake	asphalt lake
*[IMGVR_UViG_3300035494_000080|3300035494|Ga0376447_000328; IMG/VR]	n/a	2007	Trinidad and Tobago: La Brea, Pitch Lake	asphalt lake
[IMGVR_UViG_3300035493_000056|3300035493|Ga0376446_000291; IMG/VR]	VC_18_0	2007	Trinidad and Tobago: La Brea, Pitch Lake	asphalt lake
[IMGVR_UViG_3300042259_000043|3300042259|Ga0451649_000837; IMG/VR]	VC_18_0	2019	USA: Denver, Colorado	industrial wastewater
[IMGVR_UViG_3300042267_000060|3300042267|Ga0451650_000569; IMG/VR]	VC_18_0	2019	USA: Denver, Colorado	industrial wastewater
*[IMGVR_UViG_3300042269_000097|3300042269|Ga0451652_000962; IMG/VR]	n/a	2019	USA: Denver, Colorado	industrial wastewater
[IMGVR_UViG_3300033142_001157|3300033142|Ga0366826_1000308; IMG/VR]	VC_18_0	unknown (added to database 2019)	Mexico: Gulf of California	marine sediment
[†][IMGVR_UViG_3300002034_000022|3300002034|BBAY58_10000268; IMG/VR]	Outlier	unknown (added to database 2013)	Australia: Sydney, Bare Island	red algae

* Viral contig excluded from vConTACT2 due to >99% similarity to the entry directly above it in the table.

** Viral contigs identified by blastp but excluded from phage search due to contig lengths.

[†] Viral contig encodes VqmAφ-Qtip but does not cluster with VP882-like phages.

Table S2. Strains and plasmids used in this study.

Bacterial Strains<br>Parent Strain	Identifier	Genotype	Reference
V. cholerae C6706	BB-Vc0090	O1 El Tor clinical isolate (wildtype)	(2)
	BB-Vc0325	Δ*tdh* Δ*lacZ*::P$_{vqmR}$-*luxCDABE*	(3)
	BB-Vc0328	Δ*tdh* Δ*vqmA* Δ*lacZ*::P$_{vqmR}$-*luxCDABE*	(3)
	BB-Vc0820	Δ*tdh* Δ*vqmA* Δ*lacZ*::P$_{vqmR}$-*luxCDABE*<br>Δ*vc1807*::P$_{bad}$-*vqmA-specR*; Spec^{R}	(4)
BB-Vc0325	FJS-S1644	/pXBCm-P$_{bad\text{-}riboswitch}$-*gfp*	This study
	FJS-S1730	/pXBCm-P$_{bad\text{-}riboswitch}$-*tdh*^{Vp}-*FLAG*	This study
	FJS-S1731	/pXBCm-P$_{bad\text{-}riboswitch}$-*tdh*^{Vv}-*FLAG*	This study
	FJS-S1732	/pXBCm-P$_{bad\text{-}riboswitch}$-*tdh*^{Vc}-*FLAG*	This study
	FJS-S1733	/pXBCm-P$_{bad\text{-}riboswitch}$-*tdh*^{Sa}-*FLAG*	This study
	FJS-S1734	/pXBCm-P$_{bad\text{-}riboswitch}$-*tdh*^{Se1}-*FLAG*	This study
	FJS-S1735	/pXBCm-P$_{bad\text{-}riboswitch}$-*tdh*^{Se2}-*FLAG*	This study
	FJS-S1736	/pXBCm-P$_{bad\text{-}riboswitch}$-*tdh*^{C6706}-*FLAG*	This study
BB-Vc0328	FJS-S1716	/pXBCm-P$_{bad\text{-}riboswitch}$-*gfp*	This study
	FJS-S1717	/pXBCm-P$_{bad\text{-}riboswitch}$-*vqmA*φ^{1}-*FLAG*	This study
	FJS-S1718	/pXBCm-P$_{bad\text{-}riboswitch}$-*vqmA*φ^{2}-*FLAG*	This study
	FJS-S1719	/pXBCm-P$_{bad\text{-}riboswitch}$-*vqmA*φ^{3}-*FLAG*	This study
	FJS-S1720	/pXBCm-P$_{bad\text{-}riboswitch}$-*vqmA*φ^{4}-*FLAG*	This study
	FJS-S1721	/pXBCm-P$_{bad\text{-}riboswitch}$-*vqmA*φ^{5}-*FLAG*	This study
	FJS-S1722	/pXBCm-P$_{bad\text{-}riboswitch}$-*vqmA*φ^{6}-*FLAG*	This study
V. parahaemolyticus RIMD 2210633	FJS-S045	O3:K6 clinical isolate; Amp^{R} Sm^{R}	(5)
	FJS-S0063	Δ*tdh*	This study
FJS-S0063	FJS-S1614	/pEVS143-P$_{qtip}$-*luxCDABE*<br>/pXBCm-P$_{bad\text{-}riboswitch}$-*gfp*	This study
	FJS-S1687	/pEVS143-P$_{qtip}$-*luxCDABE*<br>/pXBCm-P$_{bad\text{-}riboswitch}$-*vqmA*φ^{1}-*FLAG*	This study
	FJS-S1688	/pEVS143-P$_{qtip}$-*luxCDABE*<br>/pXBCm-P$_{bad\text{-}riboswitch}$-*vqmA*φ^{2}-*FLAG*	This study
	FJS-S1689	/pEVS143-P$_{qtip}$-*luxCDABE*<br>/pXBCm-P$_{bad\text{-}riboswitch}$-*vqmA*φ^{3}-*FLAG*	This study
	FJS-S1690	/pEVS143-P$_{qtip}$-*luxCDABE*	This study

		/pXBCm-$P_{bad\text{-}riboswitch}$-*vqmAφ⁴-FLAG*	
	FJS-S1691	/pEVS143-P_{qtip}-*luxCDABE* /pXBCm-$P_{bad\text{-}riboswitch}$-*vqmAφ⁵-FLAG*	This study
	FJS-S1692	/pEVS143-P_{qtip}-*luxCDABE* /pXBCm-$P_{bad\text{-}riboswitch}$-*vqmAφ⁶-FLAG*	This study
FJS-S0045	FJS-S1693	/pEVS143-P_{qtip}-*luxCDABE* /pXBCm-$P_{bad\text{-}riboswitch}$-*gfp*	This study
	FJS-S1694	/pEVS143-P_{qtip}-*luxCDABE* /pXBCm-$P_{bad\text{-}riboswitch}$-*vqmAφ¹-FLAG*	This study
	FJS-S1695	/pEVS143-P_{qtip}-*luxCDABE* /pXBCm-$P_{bad\text{-}riboswitch}$-*vqmAφ²-FLAG*	This study
	FJS-S1696	/pEVS143-P_{qtip}-*luxCDABE* /pXBCm-$P_{bad\text{-}riboswitch}$-*vqmAφ³-FLAG*	This study
	FJS-S1697	/pEVS143-P_{qtip}-*luxCDABE* /pXBCm-$P_{bad\text{-}riboswitch}$-*vqmAφ⁵-FLAG*	This study
Vibrio vulnificus ATCC 29306		Strain CDC A1402 [P. Baumann 328]	ATCC
	FJS-S1698	/pEVS143-P_{qtip}-*luxCDABE* /pXBCm-$P_{bad\text{-}riboswitch}$-*gfp*	This study
	FJS-S1700	/pEVS143-P_{qtip}-*luxCDABE* /pXBCm-$P_{bad\text{-}riboswitch}$-*vqmAφ³-FLAG*	This study
Shewanella algae ATCC 51192		Strain IAM 14159 [OK-1]	ATCC
Salmonella enterica subsp *enterica* serovar Typhimurium ATCC 14028 (commonly referred to as 14028s)		Strain CDC 6516-60	ATCC
	FJS-S1705	/pEVS143-P_{qtip}-*luxCDABE* /pXBCm-$P_{bad\text{-}riboswitch}$-*gfp*	This study
	FJS-S1706	/pEVS143-P_{qtip}-*luxCDABE* /pXBCm-$P_{bad\text{-}riboswitch}$-*vqmAφ³-FLAG*	This study
	FJS-S1707	/pEVS143-P_{qtip}-*luxCDABE* /pXBCm-$P_{bad\text{-}riboswitch}$-*vqmAφ⁴-FLAG*	This study
E. coli TOP10		*F– mcrA Δ(mrr-hsdRMS-mcrBC) φ80lacZΔM15 ΔlacX74 recA1 araD139 Δ(ara-leu)7697 galU galK λ– rpsL(*Str^R*) endA1 nupG*	Invitrogen
	FJS-S1566	/pXBCm-$P_{bad\text{-}riboswitch}$-*gfp*	This study
	FJS-S1680	/pXBCm-$P_{bad\text{-}riboswitch}$-$tdh^{Vp}$-*FLAG*	This study
	FJS-S1681	/pXBCm-$P_{bad\text{-}riboswitch}$-$tdh^{Vv}$-*FLAG*	This study
	FJS-S1682	/pXBCm-$P_{bad\text{-}riboswitch}$-$tdh^{Vc}$-*FLAG*	This study
	FJS-S1683	/pXBCm-$P_{bad\text{-}riboswitch}$-$tdh^{Sa}$-*FLAG*	This study

	FJS-S1684	/pXBCm-P$_{bad\text{-}riboswitch}$-*tdh*^{Se1}-*FLAG*	This study
	FJS-S1685	/pXBCm-P$_{bad\text{-}riboswitch}$-*tdh*^{Se2}-*FLAG*	This study
	FJS-S1686	/pXBCm-P$_{bad\text{-}riboswitch}$-*tdh*^{C6706}-*FLAG*	This study
	FJS-S1670	/pXBCm-P$_{bad\text{-}riboswitch}$-*vqmA*φ^{1}-*FLAG*	This study
	FJS-S1671	/pXBCm-P$_{bad\text{-}riboswitch}$-*vqmA*φ^{2}-*FLAG*	This study
	FJS-S1672	/pXBCm-P$_{bad\text{-}riboswitch}$-*vqmA*φ^{3}-*FLAG*	This study
	FJS-S1673	/pXBCm-P$_{bad\text{-}riboswitch}$-*vqmA*φ^{4}-*FLAG*	This study
	FJS-S1674	/pXBCm-P$_{bad\text{-}riboswitch}$-*vqmA*φ^{5}-*FLAG*	This study
	FJS-S1675	/pXBCm-P$_{bad\text{-}riboswitch}$-*vqmA*φ^{6}-*FLAG*	This study
E. coli JKE201		λ*pir*, conjugative, diaminopimelic acid (DAP)-auxotrophic donor strain	(6)
	FJS-S1668	/pEVS143-P$_{qtip}$-*luxCDABE*	This study
	FJS-S1702	/pXBCm-P$_{bad\text{-}riboswitch}$-*gfp*	This study
	FJS-S1703	/pXBCm-P$_{bad\text{-}riboswitch}$-*vqmA*φ^{3}-*FLAG*	This study
	FJS-S1704	/pXBCm-P$_{bad\text{-}riboswitch}$-*vqmA*φ^{4}-*FLAG*	This study
E. coli S17	BB-Ec0090	/pRK2013	(7)
**Plasmids<br>Name**	**Identifier**	**Description**	**Reference**
pXBCm-P$_{bad\text{-}riboswitch}$-*gfp*	FJS-P122	Dual-control expression construct (arabinose-inducible transcription/theophylline-inducible translation) on a chloramphenicol-resistant version of the pXB300 (8) backbone; ColE1; Cm^{R}	This study
pXBCm-P$_{bad\text{-}riboswitch}$-*tdh*^{Vp}-*FLAG*	FJS-P149	C-terminal FLAG-tagged *Vibrio parahaemolyticus* 882 *tdh* allele expression plasmid; ColE1; Cm^{R}	This study
pXBCm-P$_{bad\text{-}riboswitch}$-*tdh*^{Vv}-*FLAG*	FJS-P150	C-terminal FLAG-tagged *Vibrio vulnificus* CUVETCC1 *tdh* allele expression plasmid; ColE1; Cm^{R}	This study
pXBCm-P$_{bad\text{-}riboswitch}$-*tdh*^{Vc}-*FLAG*	FJS-P151	C-terminal FLAG-tagged *Vibrio cholerae* 497778 *tdh* allele expression plasmid; ColE1; Cm^{R}	This study
pXBCm-P$_{bad\text{-}riboswitch}$-*tdh*^{Sa}-*FLAG*	FJS-P152	C-terminal FLAG-tagged *Shewanella algae* CLS1 *tdh* allele expression plasmid; ColE1; Cm^{R}	This study

pXBCm-$P_{bad\text{-}riboswitch}$-*tdh*^{Se1}-*FLAG*	FJS-P153	C-terminal FLAG-tagged *Salmonella enterica* PNUSAS042767 *tdh* allele expression plasmid; ColE1; Cm^R	This study
pXBCm-$P_{bad\text{-}riboswitch}$-*tdh*^{Se2}-*FLAG*	FJS-P154	C-terminal FLAG-tagged *Salmonella enterica* PNUSAS361348 *tdh* allele expression plasmid; ColE1; Cm^R	This study
pXBCm-$P_{bad\text{-}riboswitch}$-*tdh*^{C6706}-*FLAG*	FJS-P155	C-terminal FLAG-tagged *Vibrio cholerae* C6706 *tdh* allele expression plasmid; ColE1; Cm^R	This study
pEVS143-P_{qtip}-*luxCDABE*	pOD-58	Transcriptional fusion of the φVP882 *qtip* promoter to the *luxCDABE* operon; p15A; Km^R	(9)
pXBCm-$P_{bad\text{-}riboswitch}$-$vqmA\varphi^{1}$-*FLAG*	FJS-P143	C-terminal FLAG-tagged *Vibrio parahaemolyticus* 882 *vqmAφ* allele expression plasmid; ColE1; Cm^R	This study
pXBCm-$P_{bad\text{-}riboswitch}$-$vqmA\varphi^{2}$-*FLAG*	FJS-P144	C-terminal FLAG-tagged *Vibrio parahaemolyticus* 1171-97 *vqmAφ* allele expression plasmid; ColE1; Cm^R	This study
pXBCm-$P_{bad\text{-}riboswitch}$-$vqmA\varphi^{3}$-*FLAG*	FJS-P145	C-terminal FLAG-tagged *Vibrio parahaemolyticus* VP13206 *vqmAφ* allele expression plasmid; ColE1; Cm^R	This study
pXBCm-$P_{bad\text{-}riboswitch}$-$vqmA\varphi^{4}$-*FLAG*	FJS-P146	C-terminal FLAG-tagged *Salmonella enterica* PNUSAS042767 *vqmAφ* allele expression plasmid; ColE1; Cm^R	This study
pXBCm-$P_{bad\text{-}riboswitch}$-$vqmA\varphi^{5}$-*FLAG*	FJS-P147	C-terminal FLAG-tagged *Vibrio parahaemolyticus* VP10429 *vqmAφ* allele expression plasmid; ColE1; Cm^R	This study
pXBCm-$P_{bad\text{-}riboswitch}$-$vqmA\varphi^{6}$-*FLAG*	FJS-P148	C-terminal FLAG-tagged *Rikenellaceae* bacterium MAG302 *vqmAφ* allele expression plasmid; ColE1; Cm^R	This study
pRK2013		Conjugal helper plasmid; ColE1; Kan^R	(7)

Table S3. **Primers and synthetic DNA fragments used in this study.**

Primers Identifier	Sequence (5'→3')*	Description / Associated Construct
FJS-O549	ATGAGAGAAGATTTTCAGCCTG	Forward primer to linearize the pXBCm backbone without the *tetR*-P_{tetA} machinery / pXBCm-$P_{bad\text{-}riboswitch}$-*gfp*
FJS-O550	GCTTCAGTAGTCAGACCAG	Reverse primer to linearize the pXBCm backbone without the *tetR*-P_{tetA} machinery / pXBCm-$P_{bad\text{-}riboswitch}$-*gfp*
FJS-O551	caggactgatgctggtctgactactgaagcCTATGCTACTCCGTCAAGC	Forward primer for $P_{bad\text{-}riboswitch}$ insert from strain TND2292 (10) / pXBCm-$P_{bad\text{-}riboswitch}$-*gfp*
FJS-O552	atctgtatcaggctgaaaatcttctctcatCAAATAAAGCCAGTACAAACTG	Reverse primer for $P_{bad\text{-}riboswitch}$ insert from strain TND2292 (10) / pXBCm-$P_{bad\text{-}riboswitch}$-*gfp*
FJS-O555	GTTGAGTTGGATGCAGCACC	Forward primer to linearize the pXBCm-$P_{bad\text{-}riboswitch}$-*gfp* backbone without the *gfp* gene / all pXBCm-$P_{bad\text{-}riboswitch}$ constructs
FJS-O556	CTTGTTGTTACCTCCTTAGCAGG	Reverse primer to linearize the pXBCm-$P_{bad\text{-}riboswitch}$-*gfp* backbone without the *gfp* gene / all pXBCm-$P_{bad\text{-}riboswitch}$ constructs
FJS-O561	gcagcaccctgctaaggaggtaacaacaagATGAAAATTAAAGCACTATCAAAGCT	Forward primer for *tdh*^{Vp} insert from *V. parahaemolyticus* strain 882 / pXBCm-$P_{bad\text{-}riboswitch}$-*tdh*^{Vp}-*FLAG*
FJS-O604	agcaatttatggtgctgcatccaactcaac<u>cta</u>***cttgtcgtcatcgtctttgtagtccgg***TTCCCAATCAAGAATAACTTTGCC	Reverse primer for *tdh*^{Vp} insert from *V. parahaemolyticus* strain 882 with C-terminal FLAG tag / pXBCm-$P_{bad\text{-}riboswitch}$-*tdh*^{Vp}-*FLAG*
FJS-O563	gcagcaccctgctaaggaggtaacaacaagATGAAAATCAAAGCACTATCAAAGCT	Forward primer for *tdh*^{Vv} insert from *V. vulnificus* strain CUVETCC1 / pXBCm-$P_{bad\text{-}riboswitch}$-*tdh*^{Vv}-*FLAG*

FJS-O605	agcaatttatggtgctgcatccaactcaaccta***cttgtcgtcatcgtctttgtagtccgg***TTCCCAATCAAGAATCACTTTGC	Reverse primer for *tdh*^{Vv} insert from *V. vulnificus* strain CUVETCC1 with C-terminal FLAG tag / pXBCm-$P_{bad\text{-}riboswitch}$-*tdh*^{Vv}-*FLAG*
FJS-O565	gcagcaccctgctaaggaggtaacaacaagATGAAAATCAAAGCACTTTCAAAACTGA	Forward primer for *tdh*^{Vc} insert from from *V. cholerae* strain 497778 / pXBCm-$P_{bad\text{-}riboswitch}$-*tdh*^{Vc}-*FLAG*
FJS-O606	agcaatttatggtgctgcatccaactcaaccta***cttgtcgtcatcgtctttgtagtccgg***CTGCCAATCGAGGATAACT	Reverse primer for *tdh*^{Vc} insert from *V. cholerae* strain 497778 with C-terminal FLAG tag / pXBCm-$P_{bad\text{-}riboswitch}$-*tdh*^{Vc}-*FLAG*
FJS-O567	gcagcaccctgctaaggaggtaacaacaagATGAAAGCACTGAGTAAACTCAAGC	Forward primer for *tdh*^{Sa} insert from from *S. algae* strain CLS1 / pXBCm-$P_{bad\text{-}riboswitch}$-*tdh*^{Sa}-*FLAG*
FJS-O607	agcaatttatggtgctgcatccaactcaaccta***cttgtcgtcatcgtctttgtagtccgg***ATCCCAGCTGAGAATGACC	Reverse primer for *tdh*^{Sa} insert from *S. algae* strain CLS1 with C-terminal FLAG tag / pXBCm-$P_{bad\text{-}riboswitch}$-*tdh*^{Sa}-*FLAG*
FJS-O569	gcagcaccctgctaaggaggtaacaacaagATGAAAGCGTTATCCAAACTGA	Forward primer for *tdh*^{Se1} and *tdh*^{Se2} inserts from *S. enterica* strains PNUSAS042767 and PNUSAS361348 / pXBCm-$P_{bad\text{-}riboswitch}$-*tdh*^{Se1}-*FLAG* and pXBCm-$P_{bad\text{-}riboswitch}$-*tdh*^{Se2}-*FLAG*
FJS-O608	agcaatttatggtgctgcatccaactcaaccta***cttgtcgtcatcgtctttgtagtccgg***ATCCCAGCTCAGAATAACTTTTCC	Reverse primer for *tdh*^{Se1} and *tdh*^{Se2} inserts from *S. enterica* strains PNUSAS042767 and PNUSAS361348 with C-terminal FLAG tags / pXBCm-$P_{bad\text{-}riboswitch}$-*tdh*^{Se1}-*FLAG* and pXBCm-$P_{bad\text{-}riboswitch}$-*tdh*^{Se2}-*FLAG*
FJS-O571	gcagcaccctgctaaggaggtaacaacaagATGGAAATCAAAGCACTTTCA	Forward primer for *tdh*^{C6706} insert from pKP-443 (11) / pXBCm-$P_{bad\text{-}riboswitch}$-*tdh*^{C6706}-*FLAG*
FJS-O609	agcaatttatggtgctgcatccaactcaaccta***cttgtcgtcatcgtctttgtagtccgg***CTGCCAATCGAGGATAACTTTGC	Reverse primer for *tdh*^{C6706} insert from pKP-443 (11) with C-terminal

		FLAG tag / pXBCm-$P_{bad\text{-}riboswitch}$-*tdh*^{C6706}-*FLAG*
FJS-O573	gcagcaccctgctaaggaggtaacaacaagATGTCAATAAGCGAAGGGGATG<br>A	Forward primer for all *vqmA*φ inserts / pXBCm-$P_{bad\text{-}riboswitch}$-*vqmA*$\varphi^{X}$-*FLAG*
FJS-O599	agcaatttatggtgctgcatccaactcaaccta***cttgtcgtcatcgtctttgtagtccgg***CTT<br>GAGCAGCATCGAGAC	Reverse primer for *vqmA*$\varphi^{1\text{-}5}$ inserts with C-terminal FLAG tags/ pXBCm-$P_{bad\text{-}riboswitch}$-*vqmA*$\varphi^{X}$-*FLAG*
FJS-O602	agcaatttatggtgctgcatccaactcaaccta***cttgtcgtcatcgtctttgtagtccgg***CTT<br>GAGAAGCATCGAGACCT	Reverse primer for *vqmA*φ^{6} insert from *Rikenellaceae* MAG302 with C-terminal FLAG tag/ pXBCm-$P_{bad\text{-}riboswitch}$-*vqmA*$\varphi^{6}$-*FLAG*
**Fragments**<br>**Name**	**Sequence (5'→3')***	**Description / Associated Construct**
tdh^{Vv}	ATGAAAATCAAAGCACTATCAAAGCTAAAGCCAGAAGAAGGCATT<br>TGGATGACCGAAGTGGACAAGCCTGTTCTTGGCCACAACGATCT<br>GCTGATCAAAATTAAGAAAACCGCGATTTGTGGTACCGACGTACA<br>CATCTACAACTGGGACGAATGGTCACAAAAAACCATCCCAGTACC<br>TATGGTGGTTGGCCATGAATACGTGGGTGAAGTGGTTGGCATTG<br>GCCAAGAAGTTCGTGGTTTTGAGATCGGTGACCGCGTTTCTGGC<br>GAAGGTCACATCACTTGTGGCCACTGTCGTAACTGCCGTGGTGG<br>CCGCACGCACTTGTGCCGCAACACCATTGGTGTGGGCGTAAAC<br>CGCACAGGTTGTTTCTCTGAATACCTTGTGATCCCAGCGTTCAAC<br>GCCTTTAAAATCCCTGCAAACATCTCTGATGATCTTGCGTCTATCT<br>TCGACCCGTTTGGCAACGCAGTACACACAGCACTGTCGTTCGAT<br>CTGGTTGGTGAAGACGTACTGATCACCGGCGCTGGCCCAATCG<br>GCATCATGGCGGCTGCGGTAGCGAAGCACGTTGGTGCGCGCCA<br>CGTCGTGATCACCGATGTGAACGAATACCGTCTAGACCTCGCAC<br>GCAAAATGGGCGTGACTCGCGCGGTGAACGTTGCCGAGCAGAA<br>GCTTGACGATGTGATGGCAGAGCTAGGCATGACAGAAGGCTTCG<br>ATGTGGGCCTGGAAATGTCGGGCAACCCATCAGCATTCAACTCA<br>ATGCTGAAAACCATGAACCACGGTGGCCGTATTGCACTGCTTGG<br>CATTCCACCATCAGACATGGGCATCGATTGGAACCAAGTGATCTT<br>CAAAGGCTTGGTGATTAAAGGTATCTATGGTCGTGAAATGTTTGA<br>AACTTGGTACAAGATGGCGAGCTTGATTCAATCTGGCCTTGACCT<br>AACACCAATTATCACTCACCACTTCAAAGTGGATGATTTCCAGCA<br>AGGCTTCGACATCATGCGCAGCGGCATGTCAGGCAAAGTGATTC<br>TTGATTGGGAATAA	Synthetic insert sequence for *tdh*^{Vv} from *V. vulnificus* strain CUVETCC1 / pXBCm-$P_{bad\text{-}riboswitch}$-*tdh*^{Vv}-*FLAG*
tdh^{Vc}	ATGAAAATCAAAGCACTTTCAAAACTGAAACCAGAGCAGGGCAT<br>CTGGATGAACGAAGTGGACATGCCTGAGCTTGGCCACAACGAC<br>CTGCTGATCAAAATTAAGAAAACCGCCATTTGTGGTACTGACGTA<br>CACATTTATAACTGGGATGAGTGGTCACAAAAAACCATTCCAGTG<br>CCTATGGTAGTCGGCCATGAATATGTGGGTGAAGTGGTTGGGATT<br>GGCCAAGAAGTGCGTGGTTTCCAAATTGGTGATCGCGTTTCTGG<br>CGAAGGTCACATCACTTGTGGTCACTGCCGTAACTGCCGTGGCG<br>GCCGTACGCACCTGTGCCGTAACACCATTGGTGTGGGCGTAAAC<br>CGCACGGGTTGTTTTTCTGAATACTTAGTGATCCCAGCGTTTAAC<br>GCATTCAAGATCCCGGATGGTATTTCAGATGATCTGGCGTCTATC<br>TTCGACCCGTTTGGAAACGCTGTACACACCGCGCTTTCATTCGA	Synthetic insert sequence for *tdh*^{Vc} from *V. cholerae* strain 497778 / pXBCm-$P_{bad\text{-}riboswitch}$-*tdh*^{Vc}-*FLAG*

	CTTAGTGGGTGAAGATGTTCTGATCACCGGTGCTGGCCCAATCG<br>GCATTATGGCCGCTGCGGTTGCAAAACACGTTGGTGCGCGCCAT<br>GTGGTGATCACTGATGTGAACGAATACCGCCTCGATTTAGCTCGT<br>AAAATGGGTGTGACTCGCGCTGTGAACGTTGCGGAGCAAAATCT<br>AGAAGATGTGATGAAAGAGCTCGGCATGACCGAAGGTTTTGATG<br>TGGGCTTAGAGATGTCTGGCGTACCGAGTGCGTTTAGCGCCATG<br>CTAAAAACCATGAACCATGGTGGCCGCATCGCTCTGTTAGGTATT<br>CCACCTTCATCGATGGCGATTGATTGGAACCAGGTGATCTTCAAA<br>GGCCTTGTTATTAAAGGGATTTATGGAAGGGAAATGTTCGAAACT<br>TGGTATAAGATGGCGAGCCTCATTCAATCGGGTCTCGATATCAGC<br>CCAATTATCACTCACCACTTCAAAGTGGATGACTTCCAAAAAGGC<br>TTCGACATCATGCGCAGCGGGGCTTCCGGCAAAGTTATCCTCGA<br>TTGGCAGTAA	
tdh^{Sa}	ATGAAAGCACTGAGTAAACTCAAGCCTGAACAAGGCATCTGGAT<br>GGTAGACGCGCCCAAACCTGAAATGGGCCATAACGATCTGCTGA<br>TCAAGATTCGCAAGACCGCCATTTGTGGTACCGATGTGCATATCT<br>ACAACTGGGATGAGTGGTCACAAAAGACCATCCCGGTTCCTATG<br>GTTGTCGGCCATGAATATGTCGGCGAAGTGGTAGATATGGGTCA<br>GGAAGTTCGTGGCTTTAATATTGGTGACCGAGTGTCAGGCGAAG<br>GTCATATCACCTGTGGTCACTGCCGTAATTGCCGTGGTGGCCGC<br>ACTCATTTGTGCCGTAACACTGTGGGTGTTGGGGTTAACCGCGA<br>AGGCGCCTTCGCCGAATATCTGGTGATCCCGGCCTTCAACGCCT<br>TCAAGATCCCCGATGATATCAGCGACGATCTGGCCGCTATCTTCG<br>ACCCCTTTGGCAACGCGGTACACACTGCGCTGTCGTTTGATCTC<br>GTCGGTGAAGATGTGCTGATCACCGGAGCCGGCCCCATAGGCA<br>TTATGGCCGCCGCGGTTTGCCGCCATGTCGGTGCCCGCCACGT<br>AGTTGTCACAGATGTCAACGAATACCGTCTAGAGCTGGCGCGCA<br>AGCTGGGAGCGACCCGAGCCGTCAATGTGGCCAAGGAAAAACT<br>CGAAGATGTGATGAGTGAGCTCGGCATGACAGAGGGCTTCGAT<br>GTCGGCCTGGAAATGTCCGGTGTACCGTCAGCCTTCCACTCCAT<br>GTTGGATACCATGAACCACGGTGGCAAAATTGCCATGTTGGGGA<br>TCCCGGGTGGCGATATGGCGATAGACTGGAGCAAGGTCATCTTC<br>AAGGGGTTGGTGATAAAGGGCATCTATGGCCGGGAAATGTTTGA<br>AACCTGGTACAAGATGGCCAGTTTGATCCAGTCCGGATTGGATAT<br>TGCGCCTATTATCACCCACCATTACAAGGTGGATGACTTCCAGCA<br>GGGCTTCGATGCCATGCGCTCGGGTCAATCCGGCAAGGTCATTC<br>TCAGCTGGGATTGA	Synthetic insert sequence for *tdh*^{Sa} from *S. algae* strain CLS1 / pXBCm-$P_{bad\text{-}riboswitch}$-*tdh*^{Sa}-*FLAG*
tdh^{Se1}	ATGAAAGCGTTATCCAAACTGAAAGCGGAAGAGGGCATCTGGAT<br>GACCGACGTTCCGGAACCGGAAGTCGGCCATAACGATTTGCTGA<br>TTAAAATCCGTAAAACAGCCATCTGCGGCACTGACGTTCACATCT<br>ATAACTGGGATGACTGGTCGCAAAAAACCATCCCGGTTCCGATG<br>GTCGTGGGGCATGAATATGTCGGCGAAGTGGTCGGCATCGGTC<br>AGGAAGTGAAAGGCTTTAAAATCGGCGATCGCGTCTCCGGCGAA<br>GGTCACATCACCTGTGGTCATTGTCGCAACTGCCGTGGCGGTC<br>GTACTCACCTGTGCCGCAACACCACCGGCGTAGGCGTTAATCGT<br>CCCGGCTGCTTCGCGGAATACCTGGTCATCCCGGCATTCAATGC<br>GTTTAAAATCCCGGATAACATTTCTGATGATTTAGCCTCTATTTTC<br>GACCCGTTTGGTAATGCGGTGCATACGGCGCTGTCTTTCGATCT<br>GGTCGGCGAAGATGTACTGGTATCGGGGGCGGGGCCAATCGGC<br>GTAATGGCCGCCGCGGTGGCGAAACATGTTGGCGCGCGTCATG<br>TGGTGATTACTGACGTCAATGAATACCGTCTGGAGCTGGCGCGC<br>AAAATGGGCGTCACCCGCGCGGTCAACGTCGCGAAAGAGAGCC<br>TGAACGACGTCATGGCGGAGCTGGGAATGACCGAAGGGTTCGA<br>TGTGGGTCTGGAGATGTCCGGCGCGCCGCCGGCGTTTCGTACC<br>ATGCTGGACACCATGAATCACGGCGGTCGTATTGCGATGCTGGG<br>GATTCCGCCATCAGATATGTCTATCGACTGGACAAAAGTCATCTTT<br>AAAGGCTTGTTCATTAAAGGTATTTATGGTCGTGAGATGTTCGAA<br>ACGTGGTACAAAATGGCGGCGCTGATCCAGTCCGGTCTGGATCT<br>GTCACCGATTATCACCCATCGTTTCTCTATTGATGATTTCCAGAAA<br>GGTTTTGATGCCATGCGTTCAGGCCAGTCAGGAAAAGTTATTCT<br>GAGCTGGGATTAA	Synthetic insert sequence for *tdh*^{Se1} from *S. enterica* strain PNUSAS042767 / pXBCm-$P_{bad\text{-}riboswitch}$-*tdh*^{Se1}-*FLAG*

tdh^{Se2}	ATGAAAGCGTTATCCAAACTGAAAGCGGAAGAGGGCATCTGGAT<br>GACCGACGTTCCGGAACCGGAAGTCGGCCATAACGATTTGCTGA<br>TTAAAATCCGTAAAACAGCCATCTGCGGTACTGACGTTCACATCT<br>ATAACTGGGATGACTGGTCGCAAAAAACCATCCCGGTTCCGATG<br>GTCGTGGGGCATGAATATGTTGGCGAAGTGGTCGGCATCGGTCA<br>GGAAGTGAAAGGCTTTAAAATTGGCGATCGCGTCTCCGGCGAAG<br>GTCATATCACCTGTGGTCATTGCCGCAACTGCCGTGGTGGTCGT<br>ACTCACCTGTGTCGCAACACCACCGGCGTGGGCGTCAACCGTC<br>CCGGCTGCTTCGCGGAATATCTGGTCATCCCGGCGTTCAATGCG<br>TTTAAAATCCCGGATAACATTTCTGATGATTTAGCCTCTATTTTCGA<br>CCCGTTTGGTAATGCGGTGCATACGGCGCTGTCTTTCGATCTGG<br>TCGGCGAAGATGTACTGGTATCGGGGGCGGGGCCAATCGGCGT<br>AATGGCCGCCGCGGTGGCGAAACATGTTGGCGCGCGTCATGTG<br>GTGATTACTGACGTCAATGAATACCGTCTGGAGCTGGCGCGCAA<br>AATGGGCGTCACCCGCGCGGTCAACGTCGCGAAAGAGAGCCTG<br>AACGACGTCATGGAGGAGCTGGGAATGACCGAAGGATTCGATGT<br>GGGTCTGGAGATGTCCGGCGCGCCGCCGGCGTTTCGTACCATG<br>CTGGACACCATGAATCACGGCGGTCGTATTGCGATGCTGGGGAT<br>TCCGCCATCAGATATGTCTATCGACTGGACAAAAGTTATCTTTAAG<br>GGCTTGTTCATTAAAGGTATTTATGGTCGTGAGATGTTCGAAACG<br>TGGTACAAAATGGCGGCGCTGATCCAGTCCGGTCTGGATCTGTC<br>ACCGATTATCACCCATCGTTTCTCTATTGATGATTTCCAGAAAGGT<br>TTTGACGCCATGCGTTCAGGCCAGTCAGGAAAAGTTATTCTGAG<br>CTGGGATTAA	Synthetic insert sequence for tdh^{Se2} from *S. enterica* strain PNUSAS361348 / pXBCm-$P_{bad\text{-}riboswitch}$-$tdh^{Se2}$-*FLAG*
$vqmA\varphi^{2}$	ATGTCAATAAGCGAAGGGGATGATGCTTACATCCGCTCGTTGATT<br>CATTTTTTTGGCAATCAACCGGATCCGTGGGGCATCAAGGACAC<br>CAAGTCGGTGTTCATCTATGCAAACCAGCCCTTTCGAGAGTTAGT<br>CGGTATGAAGAACCGCAACGTGGAAGGACTTACCGACGCTGATA<br>TGGATTGCGAAACTGCGGCCTTTGCCGACTCCTTTCAGGCCCAA<br>GATAGGCTGGTCGAGCAAGGCCGGGAGAAGAAAATCGTCCTGG<br>ACGTACACCCCTACGCGAATGGTTGGCGCGTTTTCACTTTCACC<br>AAGACCCCTCTCATCATGCCGTCCGGACGTGTGGCCGGCACCA<br>TTTTCCACGGACAAGACCTGACTGACACGGCTGGCCGCATCGA<br>GCCTGCAGTGGTTGAGCTGCTGCTGCCTTCCAGTGGCCAGGCT<br>GGATCCTTCGAGACCAATGTGGTCGGTCTCAACTTGACCGAACG<br>CGAGGAACTGGTGCTGTTCTTCCTGCTTCGTGGCCGAACGGCC<br>AAGGATATCGCTGGCATGCTGGGGCGCTCTCCCCGCACCATCGA<br>ACACGCTATCGAGCGCATCCGCAACAAATTCGGTGCTGGCAACA<br>AGCGGGAGCTCATCGATATGGCCATGTCCAAGGGTTATTACAGC<br>ATGGTGCCAAAAGCCCTGTTTCACACACAGGTCTCGATGCTGCT<br>CAAGTAG	Synthetic insert sequence for $vqmA\varphi^{2}$ from *V. parahaemolyticus* strain 1171-97 / pXBCm-$P_{bad\text{-}riboswitch}$-$vqmA\varphi^{2}$-*FLAG*
$vqmA\varphi^{3}$	ATGTCAATAAGCGAAGGGGATGATGCTTACATCCGCTCGTTGATT<br>CATTTTTTTGGCAATCAACCGGATCCGTGGGGCATCAAGGACAC<br>CAAGTCGGTGTTCATCTATGCAAACCAGCCCTTTCGAGAGTTAGT<br>CGGTATGAAGAACCGCAATGTGGAAGGACTTACCGACGCCGATA<br>TGGATTGCGAAACTGCGGCCTTTGCCGACTCCTTTCAGGCCCAA<br>GATAGGCTGGTCGAGCAAGGCCGGGAGAAGAAAATCGTCCTGG<br>ACGTACACCCCTACGCGAATGGTTGGCGCGTTTTCACGTTCACC<br>AAGACCCCTCTCATCATGCCGTCCGGACGTGTGGCCGGCACCA<br>TTTTCCACGGACAAGACCTGACTGACACTGCTGGCCGCATCGAG<br>CGTGCAGTGGTTGACCTGCTGCTGCCTTCCAGTGGCCAGGCTG<br>GATCCTTCGAGACCAATGTGGTCGGTCTCAACTTGACCGAACGC<br>GAGGAGCTGGTGCTGTTCTTCCTGCTTCGTGGCAGAACAGCCA<br>AGGATATCGCTGGCATGCTGGGGCGCTCTCCCCGCACCATCGAA<br>CACGCTATCGAGCGCATCCGCAACAAATTCGGTGCTGGCAACAA<br>GCGGGAGCTCATCGATATGGCCATGTCCAAGGGTTATTACACCAT<br>GGTGCCAAAAGCCCTGTTTCACACACAGGTCTCGATGCTGCTCA<br>AGTAG	Synthetic insert sequence for $vqmA\varphi^{3}$ from *V. parahaemolyticus* strain VP13206 / pXBCm-$P_{bad\text{-}riboswitch}$-$vqmA\varphi^{3}$-*FLAG*
$vqmA\varphi^{4}$	ATGTCAATAAGCGAAGGGGATGATGCTTACATCCGCTCGTTGATT<br>CATTTTTTTGGCAATCAACCGGATCCGTGGGGCATCAAGGACAC<br>CAAGTCGGTGTTCATCTATGCAAACCAGCCCTTTCGAGAGTTAGT<br>CGGTATGAAGAACCGCAATGTGGAAGGACTTACCGACGCCGATA	Synthetic insert sequence for $vqmA\varphi^{4}$ from *S. enterica* strain PNUSAS042767 /

	TGGATTGCGAAACTGCGGCCTTTGCCGACTCCTTTCAGGCCCAA<br>GATAGGCTGGTCGAGCAAGGCCGGGAGAAGAAAATCGTCCTGG<br>ACGTACACCCCTACGCGAATGGTTGGCGCGTTTTCACGTTCACC<br>AAGACCCCTCTCATCATGCCGTCCGGACGTGTGGCCGGCACCA<br>TTTTCCACGGACAAGACCTGACTGACACGGCTGGCCGCATCGA<br>GCGTGCAGTGGTTGAGCTGCTGCTGCCTTCCAGTGGCCAGGCT<br>GGATCCTTCGAGACCAATGTGGTCGGTCTCAACTTGACCGAACG<br>CGAGGAGCTGGTGCTGTTCTTCCTGCTTCGTGGCCGAACGGAC<br>AAGGATATCGCTGGCATGCTGGGGCGCTCTCCCCGCACCATCGA<br>ACACGCTATCGAGCGCATCCGCAACAAATTCGGTGCTGGCAACA<br>AGCGGGAGCTCATCGATATGGCCATGTCCAAGGGTTATTACACCA<br>TGGTACCAAAAGCCCTGTTTCACACACAGGTCTCGATGCTGCTC<br>AAGTAG	pXBCm-P$_{bad\text{-}riboswitch}$-<br>*vqmAφ^{4}-FLAG*
vqmAφ^{5}	ATGTCAATAAGCGAAGGGGATGATGCTTACATCCGCTCGTTGATT<br>CATTTTTTTGGCAATCAACCGGATCCGTGGGGCATCAAGGACAC<br>CAAGTCGGTGTTCATCTATGCAAACCAGCCCTTTCGAGAGTTAGT<br>CGGTATGAAGAACCGCAATGTGGAAGGACTTACCGACGCCGATA<br>TGGATTGCGAAACTGCGGCCTTTGCCGACTCCTTTCAGGCCCAA<br>GATAGGCTGGTCGAGCAAGGCCGGGAGAAGAAAATCGTCCTGG<br>ACGTACACCCCTACGCGAATGGTTGGCGCGTTTTCACGTTCACC<br>AAGACCCCTCTCATCATGCCGTCCGGACGTGTGGCCGGCACCA<br>TTTTCCACGGACAAGACCTGACTGACACTGCTGGCCGCATCGAG<br>CGTGCAGTGGTTGACCTGCTGCTGCCTTCCAGTGGCCAGGCTG<br>GATCCTTCGAGACCAATGTGGTCGGTCTCAACTTGACCGAACGC<br>GAGGAGCTGGTGCTGTTCTTCCTGCTTCGTGGCAGAACAGCCA<br>AGGATATCGCAGGCATGCTGGGGCGCTCTCCCCGCACCATCGA<br>ACACGCTATCGATCGCATCCGCAACAAATTCGGTGCTGGCAACA<br>AGCGGGAGCTCATCGATATGGCCATGTCCAAGGGTTATTACACCA<br>TGGTGCCAAAAGCCCTGTTTCACACACAGGTCTCGATGCTGCTC<br>AAGTAG	Synthetic insert sequence for *vqmAφ^{5}* from *V. parahaemolyticus* strain VP10429/<br>pXBCm-P$_{bad\text{-}riboswitch}$-<br>*vqmAφ^{5}FLAG*
vqmAφ^{6}	ATGTCAATAAGCGAAGGGGATGATGCTTACATCCGCTCGTTGATT<br>CATTTTTTTGGCAATCAACCGGATCCGTGGGGCATCAAGGACAC<br>CAAGTCGGTGTTCATCTATGCAAACCAGCCCTTTCGAGAGTTAGT<br>CGGTATGAAGAACCGCAATGTGGATTGCGAAACTGCGGCCTTTG<br>CCGACTCCTTTCAGGCCCAAGATAGGCTGGTCGAGCAAGGCCG<br>GGAGAAGAAAATCGTCCTGGACGTACACCCCTACGCGAATGGTT<br>GGCGCGTTTTCACTTTCACCAAGACCCCTCTCATCATGCCGTCC<br>GGACGTGTGGCCGGCACCATTTTTCACGGACAAGACCTGACTG<br>ACACGGCTGGCCGCATCGAGCGTGCAGTGGTTGACCTGCTGCT<br>GCCTTCCAGTGGCCAGGCTGGATCCTTCGAGACCAATGTGGTC<br>GGTCTCAACTTGACTGAACGCGAGGAGCTGGTGCTGTTCTTCCT<br>GCTTCGTGGCCGAACGGCCAAGGATATCGCTGGCATGCTGGGG<br>CGCTCTCCCCGCACCATCGAACACGCTATCGAGCGCATCCGCAA<br>CAAATTCGGTGCTGGCAACAAGCGGGAGCTCATCGATATGGCCA<br>TGTCCAAGGGTTATTACACCATGGTGCCAAAAGCCCTGTTTCACA<br>CACAGGTCTCGATGCTTCTCAAGTAG	Synthetic insert sequence for *vqmAφ^{6}* from *Rikenellaceae* MAG302 /<br>pXBCm-P$_{bad\text{-}riboswitch}$-<br>*vqmAφ^{6}-FLAG*

* Lowercase letters represent homologous overlaps for Fast Cloning. Bold/italic lowercase letters represent inserted C-terminal FLAG tag sequences. Underlined lowercase letters represent STOP condons.

SUPPLEMENTAL REFERENCES

1. Lan S-F, Huang C-H, Chang C-H, Liao W-C, Lin I-H, Jian W-N, Wu Y-G, Chen S-Y, Wong H. 2009. Characterization of a New Plasmid-Like Prophage in a Pandemic *Vibrio parahaemolyticus* O3:K6 Strain. *Appl Environ Microbiol* 75:2659–2667.

2. Thelin KH, Taylor RK. 1996. Toxin-coregulated pilus, but not mannose-sensitive hemagglutinin, is required for colonization by *Vibrio cholerae* O1 El Tor biotype and O139 strains. *Infect Immun* 64:2853–2856.

3. Mashruwala AA, Bassler BL. 2020. The *Vibrio cholerae* Quorum-Sensing Protein VqmA Integrates Cell Density, Environmental, and Host-Derived Cues into the Control of Virulence. *mBio* 11:10.1128/mbio.01572-20.

4. Mashruwala AA, Decker K, Fei C, Valastayan J, Bassler BL. 2025. A transcription factor-sRNA-mediated double-negative feedback loop confers pathogen-specific control of quorum-sensing genes. *BioRxiv Prepr Serv Biol* 2025.08.22.671807.

5. Makino K, Oshima K, Kurokawa K, Yokoyama K, Uda T, Tagomori K, Iijima Y, Najima M, Nakano M, Yamashita A, Kubota Y, Kimura S, Yasunaga T, Honda T, Shinagawa H, Hattori M, Iida T. 2003. Genome sequence of *Vibrio parahaemolyticus*: a pathogenic mechanism distinct from that of *V. cholerae*. *Lancet* 361:743–749.

6. Harms A, Liesch M, Körner J, Québatte M, Engel P, Dehio C. 2017. A bacterial toxin-antitoxin module is the origin of inter-bacterial and inter-kingdom effectors of *Bartonella*. *PLoS Genet* 13:e1007077.

7. Ditta G, Stanfield S, Corbin D, Helinski DR. 1980. Broad host range DNA cloning system for gram-negative bacteria: construction of a gene bank of *Rhizobium meliloti*. *Proc Natl Acad Sci* 77:7347–7351.

8. Bina XR, Wong EA, Bina TF, Bina JE. 2014. Construction of a tetracycline inducible expression vector and characterization of its use in *Vibrio cholerae*. *Plasmid* 76:87–94.

9. Duddy OP, Silpe JE, Fei C, Bassler BL. 2023. Natural silencing of quorum-sensing activity protects *Vibrio parahaemolyticus* from lysis by an autoinducer-detecting phage. *PLoS Genet* 19:e1010809.

10. Dalia TN, Chlebek JL, Dalia AB. 2020. A modular chromosomally integrated toolkit for ectopic gene expression in *Vibrio cholerae*. *Sci Rep* 10:15398.

11. Papenfort K, Silpe JE, Schramma KR, Cong J-P, Seyedsayamdost MR, Bassler BL. 2017. A *Vibrio cholerae* autoinducer–receptor pair that controls biofilm formation. *Nat Chem Biol* 13:551–557.
